# From Microalgal Biomass to Products: Downstream Processing Technology Gaps and the Road to Commercial Diversification

**DOI:** 10.3390/microorganisms14071393

**Published:** 2026-06-24

**Authors:** Tillmann M. Peest, Nikolaus I. Stellner, S. Viswanathan, Raymond Lau, Daniel Garbe, Thomas B. Brueck

**Affiliations:** 1 Werner Siemens-Chair for Synthetic Biotechnology, TUM School of Natural Sciences, Technical University of Munich, Lichtenbergstr. 4, 85748 Garching, Germany; 2 TUM CREATE Ltd., 1 Create Way, #10-02 CREATE Tower, Singapore 138602, Singapore; 3 Nanyang Business School, Nanyang Technological University, 50 Nanyang Avenue, Singapore 639798, Singapore; 4 School of Chemistry, Chemical Engineering and Biotechnology, Nanyang Technological University, 62 Nanyang Drive, Singapore 637459, Singapore; 5 TUM-AlgaeTec Center, Technical University of Munich, 82024 Taufkirchen, Germany

**Keywords:** microalgae, microbial cell factories, biorefinery, downstream processing, techno-economic analysis, process integration

## Abstract

Commercially mature products obtained by fractionation or extraction of phototrophic microalgal biomass remain concentrated in four categories: whole-cell *Spirulina*/*Chlorella*, C-phycocyanin, astaxanthin, and DHA-rich oils. Little diversification of these fractionated, mid-tier products has followed the decline in upstream costs. Whole-cell feed and live-culture markets, agricultural biostimulants, and fermentation-derived ingredients are commercially active but lie outside this phototrophic downstream-processing scope. Reported open-pond biomass production costs have fallen from ~US$10 kg^−1^ in the 1990s to sub-US$1 kg^−1^ nth-plant projections, yet no substantial product diversification has occurred. This review brings together three complementary lines of evidence: a bibliometric analysis of 1995–2025 publications showing that downstream fractionation, biorefinery, and integrated process design account for only 9.3% of food-core microalgal research; institutional surveys documenting the same four dominant categories across Europe, China, and global markets; and a meta-analysis of 53 whole-biomass cost rows from 16 techno-economic assessments. These sources indicate consistently that downstream processing is a necessary, though not sole, constraint on commercial diversification. A four-tier unit-operation roadmap is proposed-cell disruption at commodity energy cost, fractionation with functional ingredient preservation, decolorization and desalting at food-ingredient unit cost, and standardized transferable workflows-each linked to a quantitative threshold and to the product categories it would unlock. Closing the microalgal processing technology gap now depends less on demonstrating feasibility than on meeting these thresholds.

## 1. Introduction—Microalgae as Microbial Cell Factories

Microalgae are photosynthetic microbial cell factories that have been investigated for more than five decades as producers of food, feed, biofuels, pigments, and high-value bioactive compounds [1,2]. Their attractiveness rests on autotrophic growth on minimal mineral media, protein contents that commonly reach 40–70% of dry matter in the principal commercial genera such as *Spirulina* and *Chlorella* [3], and photosynthetic conversion efficiencies that can exceed those of terrestrial crops [4]. These production-centred features have positioned microalgae as a candidate sourcing platform for sustainable proteins, lipids, and pigments at a moment when demand for non-animal food and feed ingredients is increasing due to supply chain uncertainties and climate changes effects on conventional agriculture [3,5,6].

This review concerns phototrophic microalgae and cyanobacteria grown for biomass; heterotrophic and fermentation-based routes are discussed only as comparators, not as the subject of the processing analysis. ‘Microalgae’ is used in the commercial sense and includes *Arthrospira*/*Spirulina*, following common industry and FAO usage.

### 1.1. Whole-Biomass Protein Perspective

Beyond their established role in supplements and pigments, microalgae have attracted increasing attention as sources of food-grade protein [7,8]. Their attractiveness is linked to their potentially high areal productivity [9], high protein content [10], and their potential as sources of health-promoting compounds.

**Figure 1:**
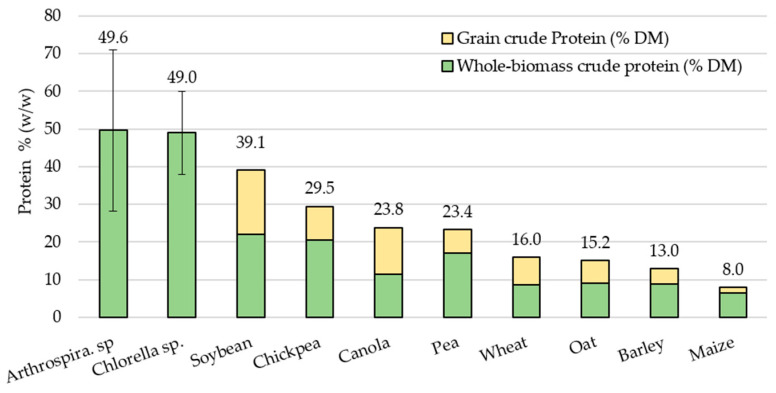
Whole-biomass versus grain-associated protein content in microalgae and crop-based protein systems. Bars show crude protein content on a dry-matter basis. For microalgae, values represent the entire harvested biomass. For crops, green indicates whole-plant biomass protein, while the yellow extension indicates grain protein. The comparison highlights that crop seeds can be protein-rich, but total plant biomass protein is lower when non-grain tissues are included. Microalgae, by contrast, produces protein-rich biomass directly at the cellular level. For crops, the grain fraction remains the relevant feedstock for food-protein processing; the whole-plant comparison is included only to illustrate different downstream starting points. Grain protein values were derived from [11] and microalgae protein values from [3].

In the context of protein generation, microalgae are fundamentally different from conventional crop-based protein systems. For crop systems the economically relevant feedstock for protein processing is typically the harvested grain fraction, not whole-plant biomass; the comparison in Figure 1 illustrates different starting points for protein supply, not a proposal to process whole-plant crop material. By contrast, microalgae generate protein-rich biomass directly at the whole-cell level, so that a much larger fraction of the produced biomass is already nutritionally relevant, and less carbon captured during photoautotrophic growth is lost to non-nutritive fractions, so a greater share of the energy fixed is valorised immediately as edible protein biomass.

### 1.2. From Crop Enrichment to Biomass Fractionation

In crop-based protein systems, processing typically starts with enrichment of the protein-bearing seed or organ fraction and only then advances toward concentrates or isolates through progressively more selective dry and wet fractionation steps, with the number of required unit operations generally increasing with product purity. For soybeans, about five preparative operations still bring the material only to soy protein flour, one further enrichment step yields soy protein concentrate, and a further six-step sequence is required to obtain soy protein isolate [12,13]. For peas, a four-step route is needed to reach isolate-like purities [14] and for the protein enrichment in chickpea, an eight-step wet-processing route is needed to yield isolate like purities [15]. In canola/rapeseed, after the defatting step at least five further operations are required [16,17,18].

This sets a benchmark of five-to-seven-unit operations from grain to protein flours and up to 11 for isolates. Microalgal biorefinery starts from a different process architecture: the first product split can already occur after harvesting as paste by solid–liquid separation or dewatering towards a powder by spray- or freeze-drying. The central task is therefore not first to enrich a protein-rich entity from the rest of the biomass, but to fractionate and valorise an already protein-containing biomass efficiently.

### 1.3. Food Relevance and Downstream Gap

At the same time, downstream processing of microalgal biomass has not yet matured into a broadly standardized industrial logic comparable to that established for conventional plant protein raw materials. Instead, the literature remains dominated by species-specific, product-specific, and step-specific solutions, with process design commonly tailored to a given biomass, target fraction, or extraction route rather than built around transferable downstream workflows [8,19,20,21]. This helps explain why microalgae are often discussed within a biorefinery framework: both technical feasibility and economic viability are more likely to improve when proteins are recovered in parallel with other valuable fractions, rather than as a stand-alone product stream [22,23,24].

While this is a great promise, the commercial record is surprisingly narrow. Microalgae have been grown at industrial scale since the 1960s [25]. The overwhelming majority of bulk commercial volume still resides in four product categories:

1. *Spirulina* and *Chlorella* sp. whole cell biomass sold as supplements;
2. C-phycocyanin extracts of varying purity grades derived mainly from *Arthrospira* sp.;
3. Natural astaxanthin extracts from *Haematococcus pluvialis*;
4. Long-chain omega-3 oils, mainly DHA-rich oils from heterotrophic *Schizochytrium* fermentation.

Microbial-cell-derived intermediate-purity products, such as protein isolates, functional polysaccharides (i.e., β-glucan), and ingredient-grade carbohydrates dominate adjacent markets, yet remain largely absent from the commercial microalgal landscape. The institutional surveys and directories examined here do not document any industrial-scale producer that co-produces multiple fractions from a single microalgal biomass stream at production volumes of tonnes of dry biomass per year or higher. Instead, some companies market lipids and the defatted raw material separately as food, feed, or biostimulants. This confirms earlier qualitative assessments that an integrated multi-product microalgal biorefinery remains a theoretical object of the techno-economic literature rather than a commercial reality [26].

This commercial narrowness coexists with substantial technical progress in cultivation. Published techno-economic assessments report a marked decline in open-pond biomass production costs, from approximately US$10 kg^−1^ dry weight in the 1990s to recent nth-plant projections below US$1 kg^−1^ AFDW [23,27,28,29,30,31]. However, this upstream cost decline has not been matched by comparable diversification of commercially established product categories.

### 1.4. Structure of the Evidence

The central question is where this gap sits and what would be required to close it. We combine three complementary, convergent lines of evidence. These lines are convergent rather than independent: bibliometric attention, commercial visibility, and techno-economic reporting can share publication and market biases. They are therefore read together, and no single line is treated as decisive on its own.

• A bibliometric analysis of 1995–2025 microalgae publications that quantifies how research attention distributes across applied, non-applied, and biorefinery-framed subsets;
• Published institutional surveys documenting hundreds of commercial enterprises worldwide;
• A curated corpus of 53 whole-biomass production-cost rows drawn from 16 peer-reviewed and grey-literature techno-economic assessments (26-row primary subset; 27-row context subset) spanning 2011–2025 (Appendix A, Table A3).

Taken together, the evidence is most consistent with downstream unit operations—rather than cultivation scale alone—being a necessary but not sufficient constraint, acting alongside regulation, sensory quality, composition, capital, and price pressure, on the delivery of mid-tier product categories at mid-tier prices. We close with a four-tier, falsifiable roadmap that names the unit-operation thresholds whose attainment would help address each of the empty commercial quadrants identified in Section 3.

## 2. The Bibliometric Signal

### Corpus and Classification

**Figure 2:**
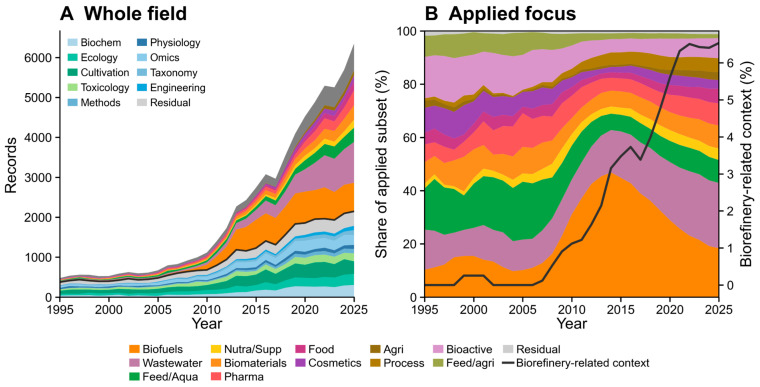
Bibliometric structure of the microalgae literature and applied topic shift. Scopus-based bibliometric mapping of the retained microalgae corpus from 1995 to 2025. (**A**) shows annual publication counts across non-applied and applied classes, with the black boundary indicating the transition from non-applied to applied categories. (**B**) resolves the applied subset by relative topic share over time and overlays the proportion of applied records containing biorefinery-related terminology. Class labels were assigned by keyword-match heuristics and should be interpreted as bibliometric estimates rather than manual expert classifications.

**Figure 3:**
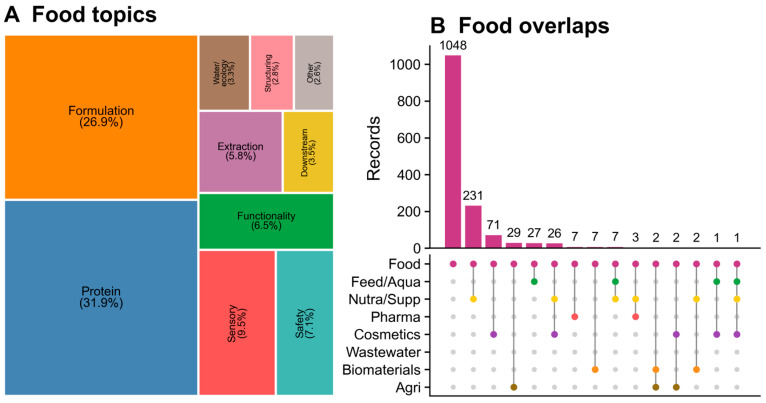
Food-focused refinement of microalgae literature. Bibliometric refinement of the food-oriented subset of the retained Scopus corpus. (**A**) shows the strict clean food-core literature as a treemap of food-related subtopics. (**B**) resolves food-centred overlap structure by combining exact overlap counts with a domain-membership matrix. Subtopic and overlap labels were assigned by keyword-match heuristics and should be interpreted as structured bibliometric estimates rather than exhaustive manual classifications.

Before examining the commercial and economic dimensions of the deficit, we asked whether the published literature itself reflects the distribution of research effort that sixty years of industrial microalgae production would lead one to expect. To do so we compiled a structured bibliometric corpus of peer-reviewed microalgae publications from 1995 to 2025 using Scopus, with category assignments, disambiguation rules, and search strings documented in Supplementary Materials S1. The resulting map covers nine non-applied categories (biochemistry, ecology, cultivation, toxicology, analytical methods, physiology, omics, taxonomy, strain engineering) and nine applied categories (food, feed/aquaculture, nutraceuticals/supplements, pharmaceuticals, biofuels, wastewater, cosmetics, agriculture, biomaterials), plus an Other/Residual bucket that is excluded from the topic-share percentages. Additionally, a biorefinery flag applied across the applied subset. Two observations emerge from this mapping in Figure 2 and Figure 3 that are directly relevant to the processing technology deficit.

First, the applied literature has expanded continuously over three decades (Figure 2A). Yet the relative weight of biorefinery-framed studies, where research explicitly invokes multi-product valorisation logic, did peak around 2015. Since then, it has declined as a share of applied output (Figure 2B). The field has not lost interest in microalgae, but integrated fractionation appears to have declined as a relative focus within applied literature.

Second, within the food-oriented subset (Figure 3A), applied output has grown faster than any other category in the past decade. The dominant topics are protein/nutrition (31.9%) and formulation/product development (26.9%). By contrast, the category that directly corresponds to the processing literature needed to support commercial food ingredients such as extraction, fractionation, biorefinery, and downstream processing/drying, accounts for only 9.3% of food-core records (127 of 1359). Work on downstream structuring, sensory/flavour refinement, and functional-performance testing occupies even smaller shares.

Under the applied-domain rule set, no food-linked records were co-classified with the wastewater or biofuel domains (Figure 3B). This is interpreted as an absence of detected cross-domain framing in the Scopus title–keyword–abstract metadata where most large-scale biorefinery knowledge is accumulated [22,24,26], rather than proof that knowledge transfer between these areas is absent. Its strongest overlap is with nutraceuticals (18.5%) and, to a lesser extent, cosmetics (7.0%)—both dominant market categories. Zero overlaps are a property of the blocker rule set rather than evidence that cross-domain literature is absent; the wastewater and biofuel anchor terms are themselves blocker terms, so any record carrying them is excluded from the clean food subset by construction. Removing those terms (targeted setting; Supplementary Materials S2) admits 377 food–wastewater and 355 food–biofuel co-classifications, but two-rater validation shows the admitted records are predominantly non-food (72.0% non-food leakage among targeted gains; 84.6% in the leave-one-out audit). The food-processing share stays within 9.0–9.8% across all blocker settings, so these counts track the rule set, not the underlying literature. Supplements and cosmetics, by contrast, justify high prices despite crude processing. One caveat: bibliometric signals measure attention, not quality, and lack of co-citation does not prove missing knowledge. The interpretation rests on convergence with commercial and economic evidence (Section 3 and Section 4), not on bibliometrics alone. A two-rater manual reclassification of a stratified 360-record sample supports these keyword labels (97.5% agreement, Cohen’s κ = 0.937; Supplementary Materials S2), with 31.2% non-food leakage in the current clean food-primary stratum (≈69% precision for the keyword food class). This Section establishes only that the literature distribution is consistent with a processing technology deficit, not that it proves one.

## 3. The Commercial Signal

### 3.1. Global Scale and Enterprise Counts

FAO-recorded microalgae cultivation reached 56,456 t in 2019 [32]. In this section, “microalgae” follows the commercial and statistical convention and includes cyanobacteria such as *Spirulina*/*Arthrospira*. The FAO-recorded tonnage was dominated by *Spirulina*/*Arthrospira*, which accounted for 56,208 t, or 99.6% of the reported total; China supplied 54,850 t, corresponding to 97.2% of FAO-recorded microalgae production [32]. These figures should be interpreted as officially recorded cultivation statistics rather than as a complete census of the global industry, since the FAO notes that substantial microalgae production may be missing from national aquaculture statistics, including production in Australia, Iceland, India, Israel, Italy, Japan, Malaysia, Myanmar, and the United States. Furthermore, those values are not directly comparable since FAO reports official aquaculture statistics, whereas the China-specific figures refer to independently estimated dry biomass for individual taxa (Table 1).

Independent industry sources therefore indicate a broader and more heterogeneous commercial landscape. In China, Gao et al. report approximately 10,000 t yr^−1^ *Spirulina* dry biomass, 2000 t yr^−1^ *Chlorella* dry biomass, and 400 t yr^−1^ *Haematococcus* dry biomass, making China the largest producer of microalgal biomass globally [25]. *Chlorella* production is clearly under-represented in FAO statistics: independent reviews estimate global *Chlorella* production at several thousand tonnes per year and identify Taiwan-based producers, including Far East Bio-Tec, among the major commercial suppliers [33]. Commercial *Dunaliella* β-carotene production was established in Australia and Israel, while DHA from heterotrophic microalgae such as *Crypthecodinium cohnii* is part of the established high-value microalgal product landscape [1].

The number of commercially active enterprises is also substantial. The JRC algae industry overview documented 548 enterprises across Europe, including 413 algae biomass producers; among these, 87 enterprises produced microalgae biomass and 213 produced *Spirulina*, with 40 enterprises producing more than one organism group [34]. Araújo et al. mapped 447 algae and *Spirulina* production units across 23 European countries with a 40% survey response rate [35]. More recently, Gallego et al. identified 146 microalgae-derived products from 66 European producers plus 49 service and technology companies [36]. Market-level estimates further place the global microalgae market at US$3.4 billion in 2020, with projected growth to US$4.6 billion by 2027 [37].

For the purposes of this review, “commercial” or “sufficiently scaled’ denotes sustained production on the order of tons of dry biomass per year, or a product placed on the open market; artisanal output (Section 3.4) is treated separately and labelled as such.

**Table 1:** Commercial data sources for the global and European microalgae landscape. The sources differ in region, reference year, metric, and basis (officially recorded cultivation tonnage, independent dry-biomass estimates, enterprise or production-unit counts, and market value), and are therefore not directly comparable. They are reported side by side to characterize the commercial landscape, not aggregated into a single figure.

Source	Region	Year	Reported Metric	Basis	Coverage	Principal Limitation
FAO aquaculture statistics [32]	Global	2019	56,456 t microalgae; *Spirulina* 56,208 t (99.6%); China 54,850 t (97.2%)	Officially recorded cultivation	Reported national aquaculture	Main FAO reviews exclude aquatic plants/algae; figure is from detailed FishStat data; omits non-reporting producers; under-reports Chlorella
Gao et al. [25,38]	China	2022	~10,000 t *Spirulina*; 2000 t *Chlorella*; 400 t *Haematococcus* yr^−1^	Dry biomass	Major Chinese producers	Independent estimate; per-taxon; methodology not fully public
JRC algae industry overview [34]	Europe (EU + Iceland/Norway)	2022	548 enterprises; 413 producers; 87 microalgae (89 units), 213 *Spirulina* (216 units); 40 produces >1 group	Enterprise/unit counts	20 EU MS + EFTA	Counts firms, not tonnage; low survey response; database-derived
Araújo et al. [35]	Europe	2021	447 algae + *Spirulina* production units across 23 countries	Production-unit counts	23 countries, 40% response	40% response → partial; counts units, not output
Gallego et al. [36]	Europe	2025	146 microalgal products from 66 producers (+49 service/tech)	Product/producer counts	66 European producers	Product inventory, not tonnage; Europe only
Market estimate [37]	Global	2020 → 2027	US$3.4 bn (2020) → US$4.6 bn (2027)	Market value (USD)	Whole microalgae market	Commercial projection, not a production census

### 3.2. The Four Established Product Categories

Throughout this review, “the four categories” refers specifically to commercially mature products recovered from phototrophic microalgal biomass by extraction or fractionation. Whole-cell, live-culture, biostimulants, and fermentation-based products are real and growing; they are treated here as adjacent categories and discussed separately, not as counterexamples to the diversification argument. Despite this industrial scale, the product portfolio of the worldwide microalgal cell factory sector remains compressed into four dominant categories that have not substantively changed since the industry was last comprehensively reviewed [1,39]. Gallego et al. found that 71% of the 146 European microalgae-derived products they identified serve food supplement, nutraceutical, or cosmetic applications, and less than 3% of European microalgal companies work on biofuels [36].

First, whole dried *Spirulina*/*Arthrospira* and *Chlorella* biomass, sold as powder or tablets, accounts for most commercial volume and roughly 40% of revenue despite comprising >95% of total algae biomass tonnage [37].

Second, C-phycocyanin extracts derived from *Arthrospira sp*., principally graded E6 to E40 by purity, are produced by approximately fifteen to twenty operators worldwide, with DIC Corporation and Zhejiang Binmei among the leading suppliers [1,36].

Third, astaxanthin-rich oleoresin fractions from *Haematococcus pluvialis* are produced by approximately ten to fifteen operators, including Solabia-Algatech, Cyanotech, Algalíf, AstaReal, and EID Parry [1,35]. These fractions are carotenoid-focused extracts, not purified astaxanthin, with composition and pigment stability dictated by biomass processing and extraction parameters [40]. This also supports the broader protein argument: pigment extraction is designed around carotenoid recovery, not preservation of native proteins. Chlorophyll-derived compounds require similar caution. In its safety assessment of an ethanolic *Phaeodactylum tricornutum* extract, EFSA noted that pheophorbide A, a chlorophyll breakdown product, was detected in all analyzed batches and that pyropheophorbide A, together with pheophorbide A, had been linked to phototoxicity in dried laver products; EFSA therefore requested specific analysis of pyropheophorbide A in the novel food. This does not imply that all pigment extracts are unsafe, but it illustrates why pigment-rich microalgal fractions require defined compositional specifications rather than being treated as generic “natural” extracts [41].

Fourth, long-chain omega-3 oils, mainly DHA-rich oils from heterotrophic *Schizochytrium* cultivated in closed fermenters, are produced by DSM-Firmenich, Corbion, Veramaris, and several Chinese operators [42].

**Figure 4:**
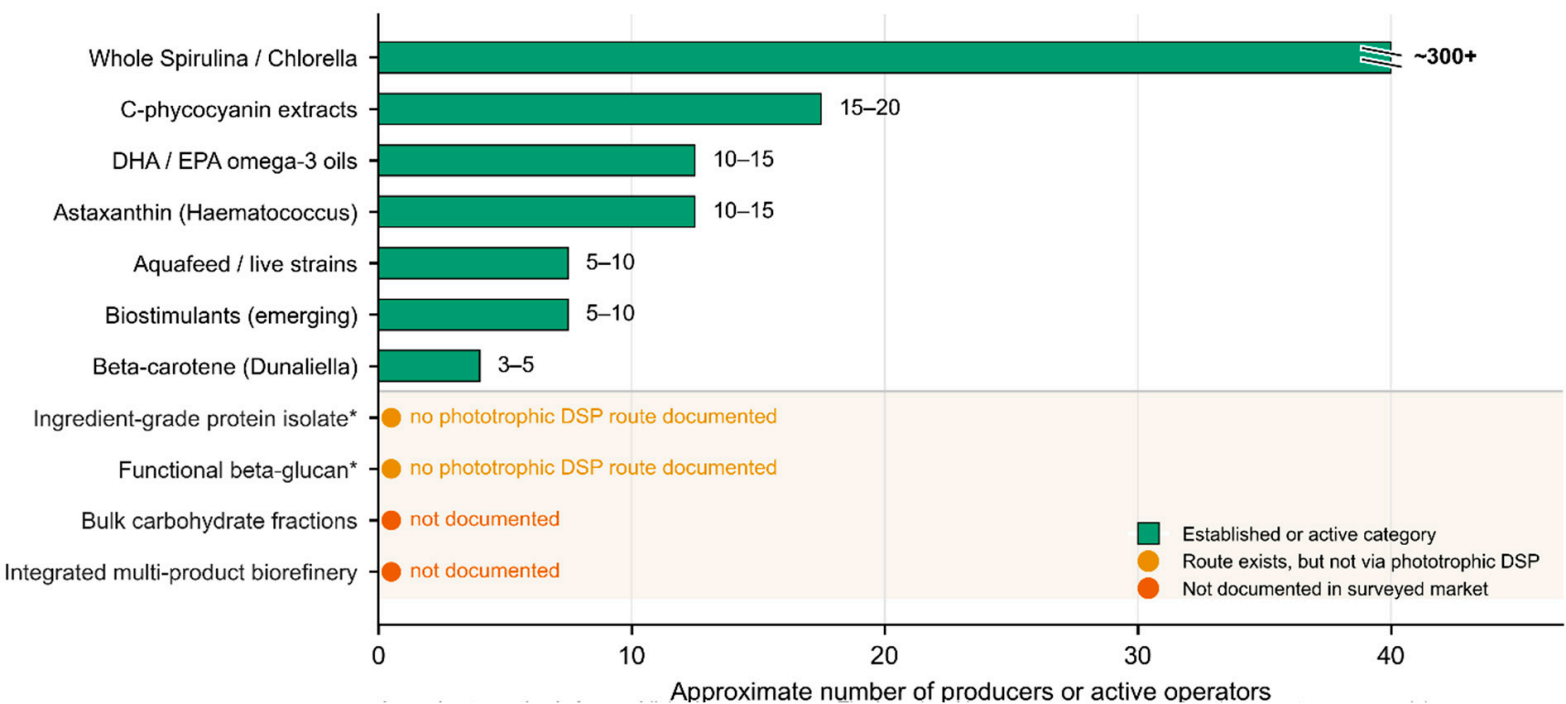
Product-category distribution of the global microalgae industry. Active categories (green) are populated by verified commercially operating producers documented in published institutional surveys [1,25,36,38,45]; empty or nascent categories (vermillion) are product types for which no commercially active producer operating from photosynthetic biomass via downstream fractionation was identified. The protein-isolate and β-glucan categories carry asterisks: commercial products exist from fermentation-based routes (Brevel, Corbion AlgaVia, Euglena paramylon) but not from phototrophic DSP. Commercial producer: an entity with at least one product offered for sale and documented in a cited survey or disclosure. Industrial scale: sustained output on the order of tonnes of dry biomass per year or above. Phototrophic downstream processing: fraction recovery from photoautotrophically grown biomass, excluding heterotrophic and mixotrophic routes. Categories were assigned as follows. Active: at least one producer with a product on sale, documented in a cited survey or disclosure. Nascent: announced, piloted, or pre-commercial activity without a verified product on sale. Undocumented: no producer operating from phototrophic biomass by downstream fractionation was found in the surveyed datasets. Asterisks mark categories where commercial products exist via fermentation routes but not via phototrophic downstream processing.

Those four categories recur across independent surveys spanning two decades and three continents (Figure 4). What these surveys do not document is more telling: the product categories that remain absent or only nascent despite extensive research attention. Three observations can be delineated. The analysis draws on published institutional and sector surveys [25,34,35,36] supplemented by company disclosures and trade-press reports cited individually below.

Aquaculture and animal feed are important adjacent markets and should not be treated as absent. Feed and aquaculture are treated as adjacent whole-cell/live-culture markets rather than purified mid-tier DSP fractions. This market is more taxonomically diverse than the human food-supplement sector and includes live or minimally processed biomass from taxa such as *Nannochloropsis*, *Tetraselmis*, *Isochrysis*, diatoms, *Chlorella*, and *Spirulina*/*Arthrospira* for hatcheries, aquafeed, pet feed, and specialty animal feed. However, these applications mostly rely on live cultures, whole cells, or minimally processed biomass rather than purified mid-tier fractions from integrated downstream fractionation. They therefore support the commercial relevance of microalgae, but do not remove the processing deficit addressed here.

First, across the institutional surveys examined, no operator is documented as selling a microalgal protein isolate from photosynthetic biomass at the purity and price point (≥75 wt% protein at USD 10–20 kg^−1^) needed to position it against mainstream plant protein isolates. Absence in these surveys is evidence that such operators are not large or public facing enough to appear in standard industry mappings; it does not rigorously exclude small or non-surveyed operators. A small number of companies, notably Aliga Microalgae, Brevel, and Corbion, are commercializing or scaling whole-cell microalgae products for the ingredient market. However, these approaches rely on heterotrophic or mixotrophic cultivation to reduce chlorophyll and associated off flavours rather than on downstream fractionation of photoautotrophically generated biomass. To our knowledge, Algama is the only company that advertises a food-grade microalgae-based egg substitute for bakery and pastry applications. No product appears to be available for purchase to date.

Second, bulk carbohydrate fractions derived from autotrophic microalgal starch or cell-wall polysaccharides are not yet commercially established. An exception exists only for selected specialty carbohydrates with biofunctional positioning. For example, Euglena Co. markets a paramylon-rich *Euglena gracilis* raw material with a reported paramylon content above 55% [43]. However, this product represents an intracellular β-1,3-glucan storage polysaccharide obtained from *Euglena* biomass, typically produced under heterotrophic or mixotrophic cultivation, rather than a carbohydrate fraction recovered from photoautotrophically generated microalgal biomass.

Third, no producer was identified in the published surveys and directories examined here that already commercializes the simultaneous co-production of protein, lipid, carbohydrate, and pigment fractions from a single microalgal biomass stream at industrial scale. This is based on the public surveys, directories, and company disclosures examined here and describes what those sources document; it does not exclude small, regional, private, or pre-commercial operators that such mappings would not capture. The integrated multi-product microalgal biorefinery, a central object of much of the TEA literature, therefore remains better established as a development target than as a verified commercial reality [23,26]. New industrial-scale initiatives indicate that this gap is being tackled. The CHITOSE Group’s MATSURI programme targets multi-product applications spanning foods, feeds, fuels, and chemicals. These efforts should be interpreted as pre-commercial platform-building, not yet as evidence of a commercially operating integrated biorefinery.

A fifth product category, agricultural and turf biostimulants, is emerging outside the four established tiers and merits acknowledgment. European sector surveys already identify microalgae-derived biostimulants and agricultural applications [36]. AlgaEnergy (Spain) restructured in 2023 to focus exclusively on microalgae-based crop inputs; Heliae’s PhycoTerra^®^ soil biostimulant is deployed on more than 10 million US acres; and the EU Fertilizing Products Regulation (2019/1009) has created the first pan-European registration pathway for microalgal biostimulants.

Recent spinouts such as Alganize illustrate a different commercial logic: microalgal cultivation is used as a biological production platform for root-stimulation and soil-health products rather than as a source of harvested food biomass [44]. In such models, value can arise from whole cell biocatalysis, extracellular metabolites, or conditioned cultivation streams, with little or no classical downstream fractionation required. This makes the category relevant as an emerging circular-bioeconomy business case, but also separates it from the protein, pigment, lipid, and carbohydrate downstream-processing deficit analyzed here.

### 3.3. Failed Diversification Ventures and Fermentation-Based Exceptions

The lower boundary of this commercial envelope is defined by three high-profile ventures that explicitly attempted product diversification beyond the four established categories but failed to reach durable commercial viability. TerraVia, formerly Solazyme, raised over US $200 million in equity and secured partnerships with Unilever, Bunge, and the United States Navy, but filed for Chapter 11 restructuring under US bankruptcy law in August 2017; its assets were later acquired by Corbion for approximately US $20 million [46]. Sapphire Energy received more than US $300 million in total funding, including US $104 million in US federal grants, but later ceased production [47]. Viridos, formerly Synthetic Genomics, received more than US $300 million from ExxonMobil under a 14-year partnership, which was terminated in 2023, before filing for Chapter 11 restructuring under US bankruptcy law in April 2025 [48]. Collectively, these ventures represent disclosed capital deployment exceeding US $600 million, backed by advanced synthetic biology platforms, government co-investment, and major industrial partners. Their failure to reach durable commercial viability indicates that diversification beyond established categories has remained commercially fragile even under unusually favourable financing and partnership conditions.

The pattern that emerges from the institutional surveys, the venture failures, and the fermentation-based exceptions is consistent. The four established product categories—whole biomass, phycocyanin, astaxanthin, and DHA/EPA—share a common downstream feature: each requires at most one or two downstream unit operations from harvested biomass (drying for whole biomass, aqueous extraction and filtration for phycocyanin, solvent or supercritical CO_2_ extraction for astaxanthin, lipid extraction for DHA). The product categories that remain absent from the commercial landscape are precisely those that would require sophisticated multi-step downstream fractionation of photosynthetically grown biomass into purified, functional fractions. The companies that succeeded at product diversification (Brevel, Corbion, Euglena) did so by abandoning the photosynthetic downstream processing chain—a correlation we report descriptively, not as evidence that phototrophic cells cannot bring commercial success. Compared with photoautotrophic cultivation, dark fermentation can achieve higher volumetric productivity but carries different sustainability trade-offs because it depends on externally supplied organic carbon. Cumulatively, these cases indicate that downstream processing is a necessary but not sufficient constraint on microalgal product diversification, acting alongside regulatory approval, sensory quality, biomass composition, and incumbent ingredient-price pressure. Alternative co-acting explanations, including regulatory gating of novel food ingredients, the sensorial constraints of photosynthetic-biomass composition limits, and mid-tier protein price compression by incumbent plant isolates, are evaluated later.

### 3.4. A Natural Benchmark: The French Paysan Spirulina Micro-Economy

The commercial evidence reviewed so far documents what is absent. The French paysan-*spirulina* sector documents what the landscape looks like when downstream processing is not a constraint and is therefore observational comparator for the processing technology-deficit claim.

The Fédération des Spiruliniers de France (FSF) groups approximately 120 adherent producers representing roughly 65% of *spirulina* farms on French territory, with the total national population estimated at 180–200 independent producers [49,50]. The federation’s 2025 techno-economic survey (69% response rate) characterizes a sector without parallel in any other microalgal product category: most farms operate at 300–900 m^2^ with a single FTE, producing 200–400 kg dry *spirulina* per year; for 52% of surveyed producers, *spirulina* is the sole source of household income; and products are sold whole as flakes, tablets, powder, or formulated foods through direct and short-circuit channels, with no fractionation step. The sector is novel but durable: 71% of farms are more than five years old, and new entrants continue to join [49].

Paysan *spirulina* production uses the minimum viable downstream chain: filter-press harvest, extrusion, and low-temperature drying below 45 °C. None of the four unit-operation tiers in Section 6 are required—mechanical cell disruption, functional fractionation, decolorization/desalting, or integrated workflows across multiple genera. In the supplement category, the commercial outcome identified in Section 3.1, Section 3.2 and Section 3.3 as absent from other microalgal product classes is the norm in this specific case. Distributed microenterprises can support full-time livelihoods at artisanal scale, with durable market penetration through direct-to-consumer channels.

The inference is not that paysan *spirulina* production can be generalized to protein isolates, polysaccharide fractions, or biorefinery co-products. The downstream chains required for those categories are qualitatively different. Rather, the French case shows that when downstream requirements are compatible with a solo operator using commodity equipment, a diversified commercial landscape can emerge without venture capital, without large industrial platforms, and without the harmonization gaps documented in Section 4. This provides observational evidence that simple downstream chains can enable commercial diversification, while broader microalgal product diversification remains constrained by downstream processing together with demand, capital availability, regulation, sensory quality, and ingredient-price pressure.

The French case is still embedded in a specific regulatory and cultural context, including the pre-novel food status of *spirulina*, a receptive consumer base, and an established artisanal food tradition. These factors likely amplify the effect, but they do not remove the central observation: commercial diversity appears when the downstream chain is simple enough to be operated reproducibly at small scale [51].

### 3.5. Alternative and Co-Acting Explanations

The interpretation advanced above—that downstream unit-operation gaps are a necessary constraint on product diversification—must be weighed against four alternative or co-acting explanations, three of which cannot be ruled out by the evidence assembled here.

#### 3.5.1. Regulatory Barriers

Novel food approval timelines under EU Regulation 2015/2283 [52] and ingredient-specific GRAS determinations in the United States remain rate-limiting for many new, microalgal food ingredients, including cellular fractions of previously approved whole algae biomass, generated by innovative processes [53,54]. Regulatory clearance is concentrated in a small set of established biomass, pigment, and oil cases-notably *Arthrospira*/*Spirulina*, *Chlorella*, *Haematococcus*-derived astaxanthin, and *Schizochytrium*-derived DHA oil-rather than extending generically to most candidate food-grade microalgae. This represents an independent commercialization barrier that downstream-processing innovation alone cannot remove. Regulation may act earlier than as a parallel cost. Where only a small set of species is authorized for food use—notably in the EU—there is limited market pull to develop fractionation capacity for the remainder, and the absence of approval can suppress downstream-processing investment before any technical threshold is reached. In this sense regulatory authorisation is a gate on whether the roadmap below is worth building for a given species, not merely an added expense.

#### 3.5.2. Price Compression from Incumbents

Pea and soy protein isolates trade at €2.5–5 kg^−1^ [46]; the €10–20 kg^−1^ window we treat as a target may itself be receding. In this scenario downstream investment could succeed technically but still fail commercially.

#### 3.5.3. Capital-Allocation Selection

The three failed diversification ventures discussed here were fuel- or commodity-first strategies, that pivoted late. Their failure may therefore reflect the well-documented difficulty of algal-fuel economics rather than downstream-processing cost alone [55,56]. Mid-tier-protein-first ventures with comparable capital intensity and world-class downstream processing have not yet been run to conclusion.

## 4. The Economic Signal-Falling Biomass Costs, Static Product Tiers

### 4.1. The TEA Corpus

**Figure 5:**
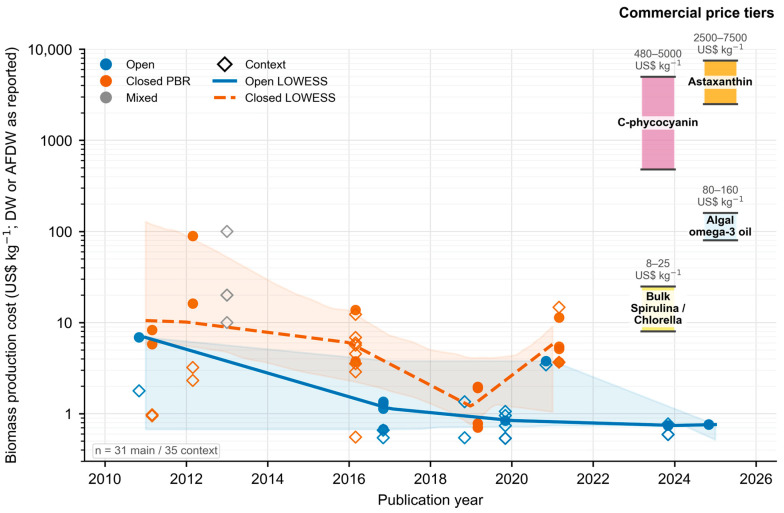
The microalgal processing–dividend gap Filled markers (n = 26) show curated primary whole-biomass production-cost estimates from peer-reviewed techno-economic studies and U.S. Department of Energy/NREL design-case reports published 2011–2025; hollow diamonds (n = 27) show context-only entries (single-pilot, optimized, derived-midpoint, or coproduct-dependent cases) and are excluded from the LOWESS trend fit and shading which are illustrative only. Marker colour denotes cultivation system: open phototrophic (raceway/pond, blue), closed phototrophic (tubular/flat-panel PBR, vermillion), and mixed/unspecified (grey). Price bands are contextual product prices, not harmonized MFSP targets. Horizontal bands show published product-price tiers: which can be found in Table A1 with its conversion factors in Table A2.

The TEA corpus assembled here is a comparative landscape, not a formal harmonization: values are reported as they appear in each source, within unit classes we treat as homogeneous, and no cash-flow, depreciation, or discount-rate normalization is attempted. Figure 5 should be read on that basis.

The commercial stasis of Section 3 would be expected in an industry whose underlying economics had not changed; the published techno-economic literature shows the opposite. Inclusion in Figure 5 required photosynthetic whole-biomass cultivation, units convertible to USD kg^−1^ (DW or AFDW), and sufficient methodological detail to assign a cultivation method system class; 53 rows from 16 studies met these criteria. Currency and mass-unit conversion factors are documented in Table A2 while the set is documented in Table A3.

### 4.2. The Biomass-Cost Trajectory

Within the set, the cost-down trajectory is unambiguous (Figure 5). Baseline estimates from the early techno-economic literature placed open-pond microalgal biomass at approximately $10,000 per tonne dry weight, roughly two orders of magnitude above any commodity price target [27]. By 2011, ref. [57] reported commercial-scale estimates of 4.95, 4.15, and 5.96 € per kg dry weight (DW) for open ponds, tubular photobioreactors, and flat-panel photobioreactors respectively, with optimized projections as low as 0.68 € per kg DW for flat-panel systems under favourable assumptions. The first real-plant TEA [58], working with a 3.8 t/y tubular facility at the University of Almería—reported 69 € per kg DW for the operating pilot and projected 12.6 € per kg DW at 200 t/y and 1.8 € per kg DW for a hypothetical 100-ha plant with free flue-gas CO_2_ and wastewater nutrients. By 2016 ref. [28] projected 3.4 € per kg DW cultivation cost at 100-ha scale in southern Spain under current technology and 0.5 € per kg DW at a ten-year projection horizon. By 2020–2022, refs. [23,29] reported 4.5–7.5 € per kg DW for 100-ha cultivation with current technology and projected sub-€1 per kg DW figures for a decade-ahead case. In the parallel North American literature, the NREL state-of-technology progression for open-pond biomass selling price declined from $1.35 kg^−1^ AFDW in 2013 ($1227 per U.S. short ton AFDW) to a 2022 design-case target of $0.54 kg^−1^ AFDW ($494 per U.S. short ton AFDW) [30,59,60].

By any standard technology-diffusion metric, upstream microalgal biomass has moved across roughly an order of magnitude in reported production cost over twenty years.

### 4.3. The Commercial Price Architecture

The commercial price architecture has not tracked this cost decline. Published market data place the four established product tiers at price points that have been broadly stable for more than a decade. Wholesale *spirulina* powder trades at approximately US$8–14 kg^−1^ FOB and chlorella at US$13–25 kg^−1^ FOB after EUR to USD conversion of the CBI 2023 ranges; these values are consistent with earlier benchmarks of US$8–10 kg^−1^ for *spirulina* and US$10–15 kg^−1^ for chlorella products [61,62]. Algal omega 3 oil trades at approximately US$80–160 kg^−1^ on a wholesale basis [1,63], and the global EPA + DHA ingredient market was valued at US$2.38 billion in 2024 [42].

Natural *Haematococcus*-derived astaxanthin, used in food-supplement/nutraceutical applications, has reported market values of approximately US$2500–7000 kg^−1^ [64] at nutraceutical grade, and food-grade C-phycocyanin trades at approximately US$480–500 kg^−1^ and above-with high-purity cosmetic and diagnostic grades reaching several thousand US$ kg^−1^ [65,66,67]. These are the product tiers that Section 3 identified; they are the tiers that can absorb the 50–60% downstream processing overhead documented in biorefinery TEAs where downstream operations consistently account for that share of total production cost [23,26,68]. This assessment is consistent with the independent finding of Gallego et al. that downstream operations are “by far the most expensive steps in microalgae production” and are normally unprofitable when only one single microalgal product is exploited. Figure 5 plots the biomass-cost trajectory against these published product-price tiers.

### 4.4. The Microalgal Processing–Dividend Gap

We use ‘processing–dividend gap’ for the combined commercial-and-processing gap between the falling upstream biomass cost and the static prices of mid-tier product classes. It is not attributed to downstream processing alone: regulation, sensory quality, biomass composition, capital availability, and competition with incumbent plant-protein isolates all operate within it. Table 2 quantifies this gap. Two points deserve explicit notification. First, the gap between upstream TEA biomass cost and retail price does not measure processing performance alone; it also measures brand margin capture, regulatory premiums, and packaging. The argument of this section is not that the entire gap is a processing failure, but rather that the existence of a large gap, together with the absence of intermediate-price product categories (Section 3), is most consistent with a processing deficit that has limited the emergence of mid-tier products in the market.

**Table 2:** Processing-relevant traits and downstream bottlenecks of selected algal production platforms. Selected algal and algal-like production systems are compared by cultivation mode, commercial product focus, cellular traits relevant to extraction, market maturity, and the dominant downstream processing bottleneck.

Taxon	Cultivation	Principal Commercial Product	Relevant Cellular Trait	Maturity	Limiting Downstream Step
*Arthrospira* */Spirulina*	Open ponds, phototrophic	Whole-cell products C-phycocyanin	Filamentous; no cellulosic wall	Mature	Pigment purification decolorization
*Chlorella*	Ponds and heterotrophic	Whole-cell products	Thick, recalcitrant cell wall	Mature	Cell disruption
*Haematococcus pluvialis*	Two-stage phototrophic	Natural astaxanthin	Thick sporopollenin cyst wall	Mature	Cyst disruption and oleoresin extraction
*Dunaliella salina*	Hypersaline ponds	β-carotene	Wall-less, fragile cells	Mature	Gentle extraction oxidative stability
*Nannochloropsis*	Phototrophic, closed/ponds	EPA, lipids, aquafeed	Robust, rigid cell wall	Emerging	Disruption + lipid extraction
*Tetraselmis*	Phototrophic	Aquaculture feed	Scaled (theca)	Emerging	Drying formulation
*Phaeodactylum tricornutum*	Phototrophic, model	EPA, fucoxanthin	Weakly silicified, pleomorphic	Nascent	Multi-product fractionation
*Schizochytrium*	Heterotrophic fermentation	DHA-rich oil	Thraustochytrid, high lipid	Mature	Oil recovery (fermentation route)
*Euglena gracilis*	Hetero/mixotrophic	Paramylon (β-1,3-glucan)	No rigid wall; pellicle	Nascent	Paramylon recovery purification

If processing costs had fallen in proportion to cultivation costs, we would expect to see either a reduction in end-consumer prices for existing products, or more plausibly the emergence of new mid-tier product categories (protein isolates, functional carbohydrates, bulk lipid fractions). Neither pattern is evident in the compiled evidence base (Table 3). Second, as noted at the outset of this section, the corpus is a comparative landscape rather than a formal harmonization. The widening space between upstream cost and commercial product architecture is what we term the microalgal processing–dividend gap. Cultivation research has generated a substantial cost dividend, but downstream translation into diversified product categories has remained limited. Section 5 and Section 6 examine where at the level of specific unit operations, the dividend would have to be spent.

**Table 3:** The microalgal processing–dividend gap. Biomass production costs (upper block) are from the curated TEA corpus (Table A3); commercial product prices (middle block) are from published peer-reviewed and institutional sources as cited. The difference between the lowest achievable biomass cost and each product-tier entry price defines the maximum tolerable processing cost per kilogram. Maximum tolerable processing cost = (lowest product-tier price) − (lowest achievable biomass cost). This is an upper envelope that must still absorb profit, logistics, packaging, regulatory compliance, quality control, and distribution; it is not a measured downstream-processing cost. The bottom block shows published estimates of the downstream processing cost share. AFDW = ash-free dry weight; DW = dry weight; FOB = free on board. † EUR → USD conversion at 2023 ECB annual average (~1.08).

	Product/Cost Category	Range/Value	Basis/Unit	Reference
Biomass production cost	Open-pond, nth-plant projection	0.54–0.76	US$ kgAFDW^−1^	[30] [31]
	Open-pond, current technology (EU, 100 ha)	3.4–6.9	US$ kgDW^−1^	[28] [23]
	Closed PBR, current technology (EU)	5.0–74	US$ kgDW^−1^	[57] [58]
Commercial product prices	Whole dried *Spirulina*, FOB	8–14 †	US$ kgDW^−1^	[62]
	Whole dried *Chlorella*, FOB	13–25 †	US$ kgDW^−1^	
	Algal omega-3 oil (DHA/EPA), wholesale	80–160	US$ kgProduct^−1^	[63] [1]
	C-phycocyanin, food-grade	480–500	US$ kgProduct^−1^	[67] [66]
	C-phycocyanin, analytical grade	1000–5000+	US$ kgProduct^−1^	[65]
	Natural astaxanthin, nutraceutical grade	2500–7000	US$ kgProduct^−1^	[64]
Processing cost share	DSP as fraction of total production cost	50–60	%	[26]
	Harvesting + dewatering, 100-ha scale	0.38–1.35	US$ kgDW^−1^	[69]

## 5. Where the Deficit Sits—Unit Operations Across the Recovery Chain

Section 2, Section 3 and Section 4 located the microalgal processing deficit at three different scales of evidence: in the literature, market, and economics. They did not, however, specify what aspects of downstream processing are missing. Protein recovery is used here as the worked example because it is the most completely documented recovery chain in the microalgal literature, with release, clarification, fractionation, and functional testing reported within the same studies. The four coupled observations that follow generalize in structure to other fractions (release is not recovery, and processing intensity trades against functional quality), but the controlling variables differ: pigment routes are governed by oxidative and thermal stability, lipid routes by solvent and phase behaviour, and polysaccharide routes by viscosity and molecular-weight control. These differences are noted where they bear on the roadmap. The protein-recovery literature provides the most thoroughly studied example. A critical reading shows that the deficit is not a single unsolved step but a set of four coupled observations recurring across studies, species, and product targets. Each observation maps onto a specific unit-operation requirement, and together they motivate the quantitative roadmap presented in Section 6.

We use the following terms consistently below: release denotes protein solubilised from disrupted cells; recovery denotes protein carried through to a defined separable fraction (clarified extract, permeate, or final product specified in each instance); extraction yield denotes released protein as a fraction of total biomass protein. Reported values are labelled with which of these they refer to, since the literature uses them interchangeably for non-comparable quantities.

### 5.1. Release Is Not Recovery

The first and most consistently reported observation is that release is not recovery. Many studies demonstrate that proteins can be liberated from microalgal biomass, but far fewer carry this material through clarification, fractionation, decolorization, desalting, and concentration into a separable, processable feed for the next unit operation [20,21]. The terminology used to report “recovery” is itself heterogeneous. Soluble protein release, aqueous extraction yield, clarified-extract recovery, permeate recovery, retentate enrichment, and final purified-product recovery are all used interchangeably and refer to non-comparable quantities. Safi et al. showed that *Nannochloropsis gaditana* biomass processing using either high-pressure homogenisation or bead milling resulted in both cases in very high cell disintegration and released roughly half the total protein at low specific energy demand [70]. By contrast, pulsed electric fields were ineffective on intact rigid-walled cells. In the subsequent sequential biorefinery study, however, the route producing the highest initial release did not deliver the highest final membrane-recovered soluble-protein fraction: HPH increased aqueous-phase protein loading, but an enzymatic route performed better after ultrafiltration/diafiltration, ultimately giving the higher permeate recovery [71]. Higher disruption severity does not automatically translate into a better overall process. This is the unit-operation-level signature of the dividend-gap problem: a step that succeeds on its own metric can still leave the chain unable to deliver a commercial-grade fraction.

### 5.2. Release Intensity Trades off Against Functional Preservation

The second observation is that release intensity trades off against functional preservation. Ursu et al. showed for *C. vulgaris* that alkaline extraction combined with mechanical disruption increased protein solubilisation, but the condition giving the highest release was not the one associated with the most favourable functional performance [72]. Other groups reached comparable conclusions for *T. suecica:* under controlled mild bead-milling conditions, they obtained a soluble fraction rich in functional proteins by exploiting the different release kinetics of proteins and carbohydrates [73]. More recent work by Ng et al. reports, that increasing NaOH concentration on *C. vulgaris* increased extraction efficiency from 6.22% to 56.23% but unsurprisingly also caused alkaline catalyzed protein hydrolysis and degradation [74]. Moreira et al. reported across freeze-thawing, enzymatic extraction, HPH, ultrasound, and pH-assisted extraction on three species that no single method was uniformly optimal: extraction route influenced both yield and protein structure, and the optima for each were different [75]. Reported recoveries must be interpreted alongside functional outcome: Ursu et al. showed 2014 that alkaline recovery of *Chlorella vulgaris* protein, while yielding 40–50% soluble protein, reduced emulsification capacity by 60% compared to native-state microfluidized extract, illustrating why recovery yield and techno-functionality cannot be substituted for one another in Tier 2 [72]. To preserve the techno-functional properties of native microalgal extracts, processing must remain within a mild operational window: conditions exceeding roughly 50–60 °C, pH 10–11 [20,26], or the presence of oxidizing agents and harsh solvents typically lead to irreversible protein denaturation and a significant loss of emulsifying and foaming performance [72,76].

Greater release does not yield a better ingredient. Downstream success depends on how upstream methods alter biomass properties, which means that any fractionation operation must be specified jointly with what it leaves behind—a constraint that single-method studies rarely meet.

### 5.3. Biomass State Is a Coupled Variable, Not a Preparative Detail

The third observation is that biomass state is a coupled variable, not a preparative detail. Halim et al. reported that complete lysis by HPH generated a heterogeneous suspension that fouled strongly during microfiltration, whereas pulsed electric field treatment with incubation preserved larger structures, produced a more permeable cake layer, and resulted in higher protein transmission together with substantially better process energy efficiency [77].

The same principle is documented in *T. suecica* [78] and across species starting from wet slurries, concentrated pastes, freeze-thawed biomass, dry powders, and freeze-dried material. None of these starting conditions are interchangeable for downstream processing [79,80]. Mear et al. reported that two biomass batches from the same supplier in different physiological states behaved very differently during bead milling [81]. Biomass history alters accessibility, aggregation, composition, and functionality before downstream processing truly begins, which means storage state and pretreatment must be treated as part of the downstream design space. The implication is operational: any standardized workflow must be benchmarked against multiple biomass states, not optimized for one.

### 5.4. Membrane Operations as the Chain-Level Test

The fourth observation is that membrane operations expose whether the preceding chain has produced a separable stream. In the screened set, membrane processes are among the few approaches that move beyond extraction toward product-relevant fractionation. Gifuni et al. proposed a three-step membrane route for *C. sorokiniana* that produced an uncolored protein fraction [79]. Soto-Sierra and Nikolov reported substantially higher recovery of soluble proteins from *Nannochloropsis* using controlled-flux ultrafiltration and diafiltration while reducing lipid carryover and chlorophyll-associated coloration [82]. Rida et al. showed that ultrafiltration in *T. suecica* crude extract simultaneously concentrated proteins and removed dissolved salts [83]. Mear et al. as well as Perez et al. add that the feed presented to a membrane is a composite material composed of proteins, carbohydrates, lipids, pigments, ash and concluded that the processability cannot be inferred from protein concentration alone [81,84]. Refs. [85,86] confirm that integrated clarification, fractionation, and polishing yield chlorophyll-free, more refined products with improved downstream performance relative to simpler routes [87,88,89].

### 5.5. Where the Deficit Sits-Unit Operations Across the Recovery Chain

We present four observations:

• Release ≠ recovery;
• Intensity ≠ preservation;
• State ≠ neutral;
• Membrane fractionation as the chain-level test.

These observations do not yet add up to a mature industrial template. However, they do define a consistent design principle: the technical and economic value of any microalgal fraction recovery improves when it is handled as one stage in an integrated valorisation sequence rather than as an isolated target from pristine biomass. The four observations also map onto four addressable unit-operation targets.

## 6. A Quantitative Roadmap for Closing the Technology Gap

### 6.1. From Biorefinery Models to Protein Recovery as a Test Case

Multiproduct cascading is already established as the economically sensible route for microalgal biorefineries. Value-chain modelling has shown that broader biomass valorisation can improve projected viability [23], while recent benchmarking and regional TEA studies extend this logic to commercial fractions and food-led business models [90,91,92].

**Figure 6:**
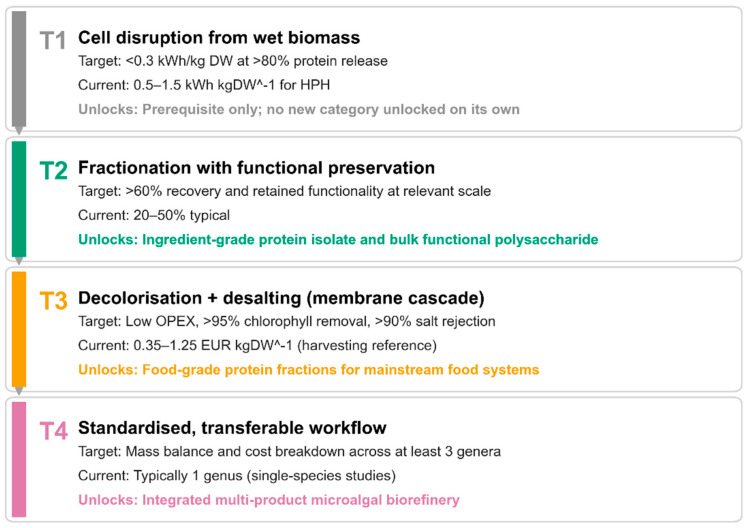
Four-tier processing roadmap with explicit dependency structure. Each tier defines a downstream unit-operation requirement, the current published state-of-the-art benchmark, and a falsifiable threshold target linked to the commercial product category it would enable. Tier 1, cell disruption from wet biomass, is a prerequisite for all subsequent steps but does not alone unlock a new product category. Tier 2, functional fractionation, and Tier 3, decolorization/desalting at food-ingredient unit cost, depend on effective disruption but can be developed in parallel. Tier 4 integrates Tiers 1–3 into a standardized transferable workflow demonstrated across at least three genera at ≥100 t y^−1^. The dependency graph therefore indicates technological coupling rather than strict serial execution. Threshold targets and current state-of-the-art values are derived from the unit-operation literature reviewed in Section 5 and Section 6 and summarized in Table 4.

In this roadmap, food-relevant protein recovery is used as a stringent test case, not as the only relevant product target. Protein fractions require cell disruption, clarification, fractionation, decolorization, desalting, and functionality retention within a food-compatible process. Since proteins are among the more fragile fractions, harsher routes for pigments, lipids, carbohydrates, or residues should be designed around protein recovery where native functionality is required [75,93]. Reviews of microalgal processing typically conclude that downstream steps require further research and development. This formulation is correct but unfalsifiable. By contrast, the four observations in Section 5, paired with the commercial and economic evidence of Section 3 and Section 4, allow a more specific statement: the deficit consists of four unit operation requirements, each with a defensible numerical target and each linked to a product category that would become commercially accessible if the target were met. These targets form a testable roadmap: each threshold can be met or missed, and the consequences for the product landscape are predictable. Table 4 specifies the four tiers; Figure 6 shows their dependency structure.

### 6.2. Tier 1—Cell Disruption at Commodity Energy Cost

The benchmark observation is that current high-pressure homogenisation typically operates at 0.5–1.5 kWh kg^−1^ DW with 50–70% protein release at conventional pressures, while pulsed electric field methods can operate well below 0.1 kWh kg^−1^ DW but achieve substantially lower release on rigid-walled [70,77,94,95,96,97,98]. Neither technology, in isolation, currently delivers more than 80% release of a target fraction at less than 0.3 kWh kg^−1^ DW from wet biomass—the energy-yield combination that would place cell disruption at parity with established industrial bioprocesses. This defines the Tier 1 threshold. Meeting it does not, by itself, unlock any new product category, but it is the prerequisite for all subsequent steps: a disruption stage that is too costly or too low-yield undermines the economics of any downstream chain, regardless of the efficiency of later operations.

### 6.3. Tier 2—Fractionation with Retained Functional Integrity

Slegers et al. showed in their industrial-scale value chain modelling that moving from single-product to multi-product extraction increases biomass utilization from 7 to 28% to >97%, but did so under modelling assumptions that have not been demonstrated experimentally at the relevant 10 kton/y reference scale [23]. Across the protein-recovery literature, reported soluble-protein recovery yields from *Chlorella*, *Tetraselmis*, and *Nannochloropsis* typically span 20–50%, and very few studies quantify the functional consequence of the recovery step [79,86,99]. We propose >60% recovery of a defined fraction with retained techno-functional properties at throughput compatible with 10 kton/y plant operation as the Tier 2 threshold. Meeting this threshold would make ingredient-grade protein isolate and mid-tier polysaccharide fractions commercially accessible.

### 6.4. Tier 3—Decolorization and Desalting at Food-Ingredient Unit Cost

Membrane cascades have been shown in lab studies to remove >95% of chlorophyll-associated coloration and to reject >90% of dissolved salts [82,83,84], but no single configuration has been benchmarked across multiple species at industrial throughput. The reference order-of-magnitude operating cost for harvesting and dewatering at 100-ha scale documented by Fasaei et al. is approximately 0.35–1.25 € kg^−1^ DW [69]; we propose that decolorization and desalting must reach a comparable unit cost, and we set the Tier 3 threshold at <0.5 € kg^−1^ DW processed biomass with the technical performance figures above. Meeting Tier 3 would provide a necessary basis for the food-grade protein gap, that the bibliometric, commercial, and protein-recovery studies together identify as the binding constraint on microalgal proteins entering mainstream food systems.

### 6.5. Tier 4—Standardized, Transferable Downstream Workflow

The fourth tier is not technical; it is coordinative. At present, no published downstream workflow has been benchmarked across at least three microalgal genera with documented mass balance and cost breakdown at a reference scale of 100 t y^−1^. The absence is not from lack of capability—the studies cited under Tiers 1–3 collectively document most of the unit operations that such a workflow would assemble—but from the absence of an integrating effort whose deliverable is a transferable template rather than a single-species single-product paper. We propose this as the Tier 4 threshold. Meeting it would help address the integrated multi-product microalgal biorefinery that the techno-economic literature has been modelling for two decades and that the commercial census of Section 3 confirms does not yet exist [23,26].

### 6.6. Falsifiability and Dependency Structure

The roadmap above is specified at the level of unit operations rather than at the level of strain engineering or cultivation because that is where the convergent evidence of Section 2, Section 3 and Section 4 places a necessary, testable constraint within a broader set of regulatory, sensory, compositional, and market-price limitations. We do not claim that each threshold can be met independently of the others; the dependency structure in Figure 6 makes explicit that Tier 2 is meaningful only if Tier 1 is in place, and Tier 4 is the integration of Tiers 1–3 across species. We do claim that the four tiers together define a falsifiable agenda. If, ten years from now, all four thresholds are met and the empty quadrants of the producer census remain empty, the explanation lies elsewhere than the processing chain and the proposed interpretation would need to be reconsidered. If, conversely, the thresholds are met and the empty quadrants begin to populate, the argument is supported.

**Table 4:** Four-tier roadmap for microalgal protein downstream processing, with an evidence-provenance (“Basis”) tag for each threshold target. Each threshold is classified individually, so that the roadmap reads as a set of falsifiable claims.

Tier	Unit Operation	Current State-of-the-Art	Threshold Target	Basis	Product Category Enabled
1	Cell disruption	HPH 0.5–1.5 kWh kg^−1^ DW, 50–70% release [70,77]; PEF ~0.05 kWh kg^−1^ DW at ~70% release on wall-deficient cells but low release on rigid walls [96].	>80% release at <0.3 kWh kg^−1^ DW from wet biomass.	Author-set target (literature-based). 80% release demonstrated for fragile-walled species (88% for *A. platensis* [98] and <0.3 kWh kg^−1^ shown for PEF [97].	Prerequisite
2	Multi-fraction recovery	20–50% soluble protein recovery; techno-functional consequences rarely quantified [79,86,99].	>60% recovery of a defined fraction with retained techno-functional properties at 10 kton y^−1^ throughput.	Author-set target (literature-based). >60% recovery is demonstrated for a defined chlorophyll-free fraction at lab scale (64 ± 1% [86]) and 10 kton y^−1^ is a modelled reference scale [23].	Ingredient-grade protein isolate; bulk functional polysaccharide
3	Decolorization and desalting	Chlorophyll removal and ≈90% salt rejection demonstrated in lab; cost not standardized across species [76,83].	Tier-3 lab performance at OPEX <0.5 € kg^−1^ DW processed biomass.	TEA back-calculation. The <0.5 € kg^−1^ ceiling is derived from the harvesting/dewatering cost band of 0.35–1.25 € kg^−1^ DW at 100-ha scale [69], placing decolorization/desalting within its lower envelope; technical performance is the secondary literature basis [82,83].	Food-grade protein fractions for mainstream food systems
4	Standardized, transferable workflow	Single-species, single-product papers dominate; no published mass-balanced workflow across multiple genera at industrial reference scale [26].	Documented mass balance + cost breakdown across ≥3 microalgal genera at ≥100 t y^−1^ reference scale.	Author-set target (coordinative). No mass-balanced multi-genera workflow exists; the closest prior work models single-product value chains across strains rather than a demonstrated, transferable workflow [23].	Integrated multi-product microalgal biorefinery

## 7. Conclusions

Upstream cultivation of microalgae has advanced substantially over the past two decades. The techno-economic evidence (Section 4) documents an order-of-magnitude decline in reported open-pond biomass production cost over twenty years, and published institutional surveys document hundreds of commercial enterprises across major production regions [25,35,38]. This cultivation progress has not been matched by comparable downstream diversification: the commercial landscape remains concentrated in the same four categories—whole dried *Spirulina* and *Chlorella*, C-phycocyanin, natural astaxanthin, and DHA omega-3 oils—that dominated it before the cultivation cost decline began; three high-profile diversification ventures with collective disclosed funding exceeding US$600 million have failed; and the few companies that have successfully diversified (Corbion AlgaVia, Brevel, Euglena) did so by abandoning photosynthetic biomass and switching to heterotrophic or mixotrophic fermentation.

The convergent evidence from bibliometric mapping, published institutional surveys of the commercial landscape, and a meta-analysis of 53 techno-economic cost rows from 16 studies identifies downstream unit operations as a necessary, currently under-resourced condition for commercial diversification. The evidence assembled here does not fully resolve whether downstream processing is a sufficient condition for diversification, or whether it partly reflects broader constraints related to photosynthetic-biomass composition, regulatory timelines, and mid-tier protein price competition. The evidence base is dominated by a small set of well-studied genera (*Chlorella, Spirulina (Arthrospira), Nannochloropsis, Tetraselmis, Haematococcus, and Schizochytrium*) and the roadmap is framed for microalgae as studied here; extension to less-studied genera remains a reasonable conjecture rather than a demonstrated result. The road ahead for implementing value-adding algal biomass fractions, such as bio- and techno-functional protein fractions into the market relies on the four tier sections outlined in Section 6 of this review. Meeting these goals under industrial-scale economic and ecological constraints will determine whether the sector can diversify and further valorise existing algal biomass using circular bio-economy principles.

## 8. Literature Search Strategy

### 8.1. Bibliometric Corpus

A reproducible rule-driven pipeline was applied to Scopus title, abstract, and author-keyword metadata for 1995–2025. The final corpus covered 1995–2025 and contained 67,195 retained peer-reviewed journal articles and reviews. The 2026 records were excluded because the retrieved 2026 subset was incomplete and lacked abstract text, making it non-comparable for title–keyword–abstract classification (2025: 6348 retained records, 6301 with abstracts; 2024: 5697/5684; 2026: 2287 retrieved, 0 with abstracts).

Retrieval used a year-wise ‘TITLE-ABS-KEY’ query combining 13 microalgae terms (*microalgae*, *micro-algae*, *microalgal*, *microalga*, *microphyte*, *Chlorella*, *Nannochloropsis*, *Dunaliella*, *Haematococcus*, *Scenedesmus*, *Tetraselmis*, *Arthrospira*, *Spirulina*). Records were filtered through a seven-step cascade (valid JSON → year extraction → deduplication → 1995–2025 window → journal aggregation type → article or review subtype → microalgae corpus boundary) with the boundary filter retaining any record that either contained one of 28 direct microalgae anchor terms or was published in one of nine whitelisted algal journals. Retained records were then classified by a hard-anchor/anchor/support scoring rulebook spanning nine applied clusters (food, nutraceuticals/supplements, pharmaceuticals, feed/aquaculture, biofuels, wastewater/environmental, cosmetics/pigments, agriculture/ biostimulants, biomaterials) and 9 non-applied clusters (biochemistry, ecology, cultivation/reactor, toxicology, analytical methods, physiology/photosynthesis, omics, taxonomy, strain engineering). Nine cross-cutting tags (biorefinery context, integrated processing, downstream processing, extraction/fractionation, protein focus, pigment focus, lipid focus, waste-to-product, cultivation coupling) were applied independently of cluster membership. Five QC flags (ambiguity, generic-primary-only, large multi-membership, food-blocker co-occurrence, source-title sensitivity) were computed per record. For downstream food-subset analysis, only records satisfying the ‘cluster_primary_clean’ gate (applied = True AND qc_ambiguous = False) were selected. The food cluster additionally requires one of 16 food-specific gate terms and is blocked by a list of 55 cross-domain terms (biofuel, wastewater, aquaculture, pharmaceutical, cosmetic, bioplastic, and related process/health/material language); this blocker design is by construction and conservatively suppresses some food-linked cross-domain research. The complete rulebook (all term lists, thresholds, blocker sets, and QC definitions), the corpus flow counts, and the panel-level annual count tables are supplied as Appendix A.

### 8.2. The Commercial Landscape

The commercial landscape analysis (Section 3, Figure 4) synthesizes published institutional surveys of the global microalgae industry, including an algae industry database ([34]; 548 enterprises across 24 countries), a European production-unit census ([35]; 447 units across 23 countries, 40% survey response rate), a European microalgal product survey ([36]; 146 products from 66 producers), FAO global aquaculture production statistics [32,100,101], and a review of Chinese microalgae industry development spanning six decades [25]. Data are presented in Table 1 and Figure 4.

### 8.3. The TEA Evidence

The TEA corpus (Figure 5; Table A3) was assembled through a structured literature search and a documented three-stage extraction pipeline. In Stage 1, we queried Scopus, Google Scholar, and the NREL publication database for peer-reviewed articles, government reports, and grey-literature techno-economic assessments reporting quantitative microalgal biomass production costs, using the terms microalg AND (techno-economic OR TEA OR production cost OR biomass cost). Entries were excluded if they reported fuel-only minimum fuel selling price (MFSP), lipid-only extraction cost, wastewater-credit-dependent values, biorefinery minimum-diesel-selling-price, or values not traceable to a primary calculation. The final dataset is a curated 53-row subset, shown in Table A3 and plotted in Figure 5. The empirical evidence assembled here is dominated by *Chlorella*, *Spirulina* (*Arthrospira*), *Nannochloropsis*, *Tetraselmis*, *Haematococcus*, and *Schizochytrium*; the roadmap is formulated for species within this envelope. Extension to less-studied genera (e.g., *Porphyridium, Galdieria*, diatoms) is a reasonable conjecture, not a demonstrated result. The corpus is also bounded to upstream whole-biomass production cost: fuel-only, lipid-only, wastewater-credit-dependent, and biorefinery values were excluded to keep the cost rows comparable, so extraction-, purification-, and formulation-specific techno-economic data are under-represented.

## Data Availability

The original contributions presented in this study are included in the article/Supplementary Material. Further inquiries can be directed to the corresponding author.

## Supplementary Materials

The following supporting information can be downloaded at: https://www.mdpi.com/article/10.3390/microorganisms14071393/s1. Supplementary Materials S1. Bibliometric classification pipeline. This supplement documents the rule-based pipeline used to classify the microalgae literature in Section 2. The corpus comprises peer-reviewed journal articles and reviews indexed in Scopus for 1995–2025 (67,195 records), classified from title, author-keyword, and abstract metadata only; no full text is used. Records are assigned to nine applied categories (food, feed/aquaculture, nutraceuticals/supplements, pharmaceuticals, biofuels, wastewater, cosmetics, agriculture, biomaterials) and nine non-applied categories under a fixed precedence ladder, with a residual “Other/Residual” bucket excluded from topic-share percentages. The food subset is further resolved into sub-topics, and a deterministic 55-term food blocker separates food-product research from the six competing applied domains. Complete term lists, the precedence ladder, the corpus-flow funnel, and all rulebooks are tabulated below; the food-blocker sensitivity and manual validation are reported separately in Supplementary S2. Supplementary Materials S2. Food-blocker sensitivity and manual validation. This supplement tests whether the food-domain results of Section 2 depend on the food-blocker rule set, and reports the manual validation of the keyword labels, both on the full 1995–2025 corpus. Section S2.1 reports the food processing/fractionation share and the food–wastewater/food–biofuel co-classification counts under four blocker settings (current 55-term, targeted 36-term, conservative 22-term, and complete removal) and a leave-one-out audit of all 55 terms. Section S2.2 reports an independent two-rater re-classification of a stratified sample of 360 records, with inter-rater agreement, Cohen’s κ, the consensus-label distribution, and per-stratum leakage rates. Together these show that the processing/fractionation share is stable across blocker settings (range 0.77 percentage points; all single-term removals within 9.2–9.6%), that the zero food–wastewater/food–biofuel counts are a property of the rule set rather than evidence of an absent literature, and that the records admitted when the blocker is lifted are predominantly non-food.

## Abbreviations

The following abbreviations are used in this manuscript:

AFDW	Ash-free dry weight
ATS	Algal turf scrubber
CAP	Combined algal processing
DHA	Docosahexaenoic acid
DSP	Downstream processing
DW	Dry weight
EPA	Eicosapentaenoic acid
FOB	Free on board
FSF	Fédération des Spiruliniers de France
GCC	Gulf Cooperation Council
GRAS	Generally recognized as safe
GWP	Green Wall Panel
HPH	High-pressure homogenisation
JRC	Joint Research Centre
LOWESS	Locally weighted scatterplot smoothing
MBSP	Minimum biomass selling price
MFSP	Minimum fuel selling price
NREL	National Renewable Energy Laboratory
OPEX	Operational expenditure
PBR	Photobioreactor
PEF	Pulsed electric field
SOT	State of technology
TEA	Techno-economic assessment/analysis
AFDW	Ash-free dry weight
ATS	Algal turf scrubber
CAP	Combined algal processing
DHA	Docosahexaenoic acid

## Appendix A

**Table A1:** Published product-price bands for microalgae-derived biomass and high-value products. The table summarizes literature- and trade-intelligence-derived price tiers used to contextualize microalgal biomass production-cost estimates in Figure 5. Prices are reported as approximate USD kg^−1^ bands for bulk *Spirulina*/*Chlorella* biomass, algal omega-3 oil, C-phycocyanin, and natural astaxanthin. These values represent product-market price ranges or reported profitability/wholesale thresholds rather than techno-economic biomass production costs. They are therefore used only as commercial context for interpreting cost competitiveness across bulk biomass, specialty ingredients, and high-value microalgal products.

Product	Tier	Low Price (USD kg^−1^)	High Price (USD kg^−1^)	Source Note	Reference
Bulk *Spirulina*/*Chlorella*, FOB	Bulk biomass	8	25	*Spirulina* €7–13 kg^−1^ and *Chlorella* €12–23 kg^−1^ FOB; converted to approximately USD 8–25 kg^−1^.	[62]
Algal omega-3 oil, wholesale	Omega-3 oil	80	160	Matos reports a current wholesale market price around USD 140 kg^−1^, with a range of USD 80–160 kg^−1^; Borowitzka reports the same range.	[1,63]
C-phycocyanin, food-grade and above	Phycocyanin	480	5000	Williams reports USD 480 kg^−1^ as a profitability threshold; Pozzuoli reports food-grade values in the few-hundred USD kg^−1^ range and high-purity material in the several-thousand USD kg^−1^ range.	[65,67]
Natural astaxanthin, nutraceutical grade	Astaxanthin	2500	7000	Natural/algae-derived astaxanthin for food-supplement and nutraceutical applications, US$2500–7000 kg^−1^.	[64]

**Table A2:** Conversion factors used to normalize reported microalgal biomass cost estimates. The table documents the currency and mass-unit conversion factors applied to convert source-reported values into USD kg^−1^. EUR-denominated values were converted using nominal year-specific exchange factors, while values reported per metric tonne or U.S. short ton were converted to kg using the corresponding mass factor. The normalization was limited to currency and unit conversion; no inflation adjustment, purchasing-power correction, or dry-weight to ash-free dry weight conversion was applied unless explicitly reported by the source.

Cost Year	Reported Source Unit	Currency/Mass Class	Currency Factor to USD	Formula Used USD kg^−1^	Reference Using Factor
2011	EUR/kg DW	EUR per kg	1.392	EUR kg^−1^ × 1.392	[57]
2012	EUR/kg DW	EUR per kg	1.286	EUR kg^−1^ × 1.286	[58]
2013	$/t DW	USD per metric tonne	1.000	USD t^−1^ × 0.001	[2]
2016	EUR/kg DW	EUR per kg	1.107	EUR kg^−1^ × 1.107	[28,102]
2017	$/ton AFDW	USD per US short ton	1.000	USD US-ton^−1^ × 0.001102	[30,59,103,104,105]
2017	EUR/kg algae	EUR per kg	1.130	EUR kg^−1^ × 1.130	[106]
2017	2014 $/metric tonne AFDW, 20% solids	USD per metric tonne	1.000	USD metric-tonne^−1^ × 0.001	[107]
2019	2014 $/US ton AFDW	USD per US short ton	1.000	USD US-ton^−1^ × 0.001102	[108,109]
2020	2016 $/US ton AFDW	USD per US short ton	1.000	USD US-ton^−1^ × 0.001102	[104,110]
2021	EUR/kg DW or EUR/kg biomass	EUR per kg	1.183	EUR kg^−1^ × 1.183	[111,112]
2024	2020 $/US ton AFDW	USD per US short ton	1.000	USD US-ton^−1^ × 0.001102	[109]
2025	$/ton AFDW	USD per US short ton	1.000	USD US-ton^−1^ × 0.001102	[31]
2025	EUR/kg dry biomass	EUR per kg	1.111	EUR kg^−1^ × 1.111	[92]

**Table A3:** Curated source-audited techno-economic cost estimates for microalgal biomass production. The table summarizes primary and context-level techno-economic estimates of microalgal biomass production costs used to support the cost-trajectory analysis. Reported costs are retained on the original source basis and additionally normalized to USD kg^−1^ for cross-study comparison. Evidence tiers distinguish primary evidence, boundary-specific primary evidence, and context/sensitivity cases. Costs differ in system boundary, cultivation system, scale, geography, biomass basis, and inclusion of harvesting, dewatering, storage, contingency, or coproduct effects; therefore, normalized values should be interpreted as comparative literature benchmarks rather than harmonized minimum selling prices.

Evidence Tier	Year	System	Case Type	Region	Scale	Reported Cost	Normalized Cost (USD kg^−1^)	Basis	Comparability Note	Ref.
Primary evidence	2012	Tubular PBR	Real plant, 3.8 t/y	EU	pilot	69.3 EUR/kg DW	89.12	DW	Real pilot plant; high cost reflects small scale.	[58]
Primary evidence	2012	Tubular PBR	Scaled plant, 200 t/y	EU	hypothetical	12.6 EUR/kg DW	16.20	DW	Scaled modelled plant scenario.	
Context/sensitivity	2012	Tubular PBR	Large plant, free flue-gas CO_2_	EU	hypothetical	2.5 EUR/kg DW	3.215	DW	Optimized/free-input scenario.	
Context/sensitivity	2012	Tubular PBR	Large plant, wastewater nutrients + free CO_2_	EU	hypothetical	1.8 EUR/kg DW	2.315	DW	Optimized/free-input scenario.	
Context/sensitivity	2013	Mixed/commercial systems	Commercial estimate: Chlorella	global	industrial	20,000 $/t DW	20.00	DW	Approximate commercial direct-cost estimate; not full TEA.	[2]
Context/sensitivity	2013	Mixed/commercial systems	Commercial estimate: Dunaliella	global	industrial	20,000 $/t DW	20.00	DW	Approximate commercial direct-cost estimate; not full TEA.	
Context/sensitivity	2013	Mixed/commercial systems	Commercial estimate: Haematococcus	global	industrial	100,000 $/t DW	100	DW	Approximate commercial direct-cost estimate; not full TEA.	
Context/sensitivity	2013	Mixed/commercial systems	Commercial estimate: Spirulina	global	industrial	10,000 $/t DW	10.00	DW	Approximate commercial direct-cost estimate; not full TEA.	
Primary evidence	2019	GWP-II flat-panel PBR	GWP-II design	US	hypothetical	1793 $/US ton AFDW	1.976	AFDW	Future/nth-plant NREL closed-PBR MBSP.	[108,110]
Primary evidence	2019	Hanging-bag PBR	Leidos hanging-bag PBR	US	hypothetical	639 $/US ton AFDW	0.704	AFDW	Future/nth-plant NREL closed-PBR MBSP.	
Primary evidence	2019	Helical tubular PBR	Helical tubular PBR	US	hypothetical	1737 $/US ton AFDW	1.914	AFDW	Future/nth-plant NREL closed-PBR MBSP.	
Primary evidence	2019	Horizontal tubular PBR	Horizontal tubular PBR	US	hypothetical	708 $/US ton AFDW	0.78	AFDW	Future/nth-plant NREL closed-PBR MBSP.	
Primary evidence	2020	Open raceway pond	FY19 SOT, ASU evaporation, unlined	US	hypothetical	764 $/US ton AFDW	0.842	AFDW	FY19 NREL SOT main unlined-pond case.	[110]
Context/sensitivity	2020	Open raceway pond	FY19 SOT, ASU evaporation, fully lined	US	hypothetical	961 $/US ton AFDW	1.059	AFDW	Alternative NREL SOT scenario; context.	
Context/sensitivity	2020	Open raceway pond	FY19 SOT, FA evaporation, fully lined	US	hypothetical	866 $/US ton AFDW	0.954	AFDW	Alternative NREL SOT scenario; context.	
Context/sensitivity	2020	Open raceway pond	FY19 SOT, FA evaporation, unlined	US	hypothetical	670 $/US ton AFDW	0.738	AFDW	Alternative NREL SOT scenario; context.	
Primary evidence	2017	Open raceway pond	2015 SOT, ATP3	US	hypothetical or SOT model	1227 $/ton AFDW	1.352	AFDW	NREL SOT benchmark; modelled open-pond MBSP.	[59]
Primary evidence	2017	Open raceway pond	2016 SOT, ABY1 performer	US	hypothetical or SOT model	1031 $/ton AFDW	1.136	AFDW	NREL SOT benchmark; modelled open-pond MBSP.	
Primary evidence	2017	Open raceway pond	2016 SOT, ATP3	US	hypothetical or SOT model	1171 $/ton AFDW	1.29	AFDW	NREL SOT benchmark; modelled open-pond MBSP.	
Context/sensitivity	2017	Open raceway pond	2020 projection	US	hypothetical	598 $/ton AFDW	0.659	AFDW	NREL projection/target; not actual SOT.	
Context/sensitivity	2017	Open raceway pond	2022 design-case target	US	hypothetical	494 $/ton AFDW	0.544	AFDW	NREL projection/target; not actual SOT.	
Context/sensitivity	2020	Open raceway pond	Delivered feedstock input, 18 wt% solids	US	hypothetical	486 $/dry US ton AFDW, delivered at 18 wt% solids	0.536	AFDW	Delivered feedstock input in CAP conversion TEA.	[104]
Primary evidence	2024	Open raceway pond	2022 harmonization, pre-storage	US	hypothetical	674$/US ton AFDW	0.743	AFDW	BT23 national weighted-average pre-storage MBSP.	[109]
Context/sensitivity	2024	Open raceway pond	2022 harmonization, after seasonal storage	US	hypothetical	701 $/US ton AFDW	0.773	AFDW	After seasonal storage; storage-boundary context.	
Primary evidence	2017	Algal turf scrubber	ATS baseline	US	hypothetical	510.65 $/metric tonne AFDW, 20% solids	0.511	AFDW	Production + harvesting to 20% solids; metric-ton conversion.	[107]
Primary evidence	2017	Open raceway pond	ORP baseline	US	hypothetical	673.65 $/metric tonne AFDW, 20% solids	0.674	AFDW	Production + harvesting to 20% solids; metric-ton conversion.	
Boundary-specific primary	2025	Greenhouse-supported raceway	ABM I, grade-A biomass, cultivation + harvesting	Greece	hypothetical	5.98 EUR/kg dry biomass	6.644	dry biomass	Cultivation + harvesting only; narrower boundary than full production.	[92]
Context/sensitivity	2017	Integrated PBR biorefinery	Multiproduct value chain, high endpoint	EU	pilot or project model	8.8 EUR/kg algae	9.944	algae/value-chain	Integrated multiproduct value-chain cost; not clean cultivation-only cost.	[106]
Context/sensitivity	2017	Integrated PBR biorefinery	Multiproduct value chain, low endpoint	EU	pilot or project model	7.1 EUR/kg algae	8.023	algae/value-chain	Integrated multiproduct value-chain cost; not clean cultivation-only cost.	
Primary evidence	2011	Flat-panel PBR	Base case, 100 ha, Dutch climate	The Netherlands	hypothetical	5.96 EUR/kg DW	8.296	DW	Modelled 100-ha base case including dewatering.	[57]
Primary evidence	2011	Horizontal tubular PBR	Base case, 100 ha, Dutch climate	The Netherlands	hypothetical	4.15 EUR/kg DW	5.777	DW	Modelled 100-ha base case including dewatering.	
Primary evidence	2011	Open raceway pond	Base case, 100 ha, Dutch climate	The Netherlands	hypothetical	4.95 EUR/kg DW	6.89	DW	Modelled 100-ha base case including dewatering.	
Context/sensitivity	2011	Flat-panel PBR	Optimized: Bonaire + PE/medium/CO_2_ assumptions	Bonaire	hypothetical	0.68 EUR/kg DW	0.947	DW	Optimized sensitivity; not a base case.	
Context/sensitivity	2011	Horizontal tubular PBR	Optimized: Bonaire + PE/medium/CO_2_ assumptions	Bonaire	hypothetical	0.7 EUR/kg DW	0.974	DW	Optimized sensitivity; not a base case.	
Context/sensitivity	2011	Open raceway pond	Optimized: Bonaire + PE/medium/CO_2_ assumptions	Bonaire	hypothetical	1.28 EUR/kg DW	1.782	DW	Optimized sensitivity; not a base case.	
Primary evidence	2016	Flat-panel PBR	Current modelled cost, 100 ha, Spain	EU/Spain	hypothetical	3.4 EUR/kg DW	3.764	DW	Current 100-ha modelled Spain case.	[28,112]
Context/sensitivity	2016	Flat-panel PBR	Future 10-year projection, Spain	EU/Spain	hypothetical	0.5 EUR/kg DW	0.553	DW	Future projection; context only.	
Primary evidence	2021	Tubular PBR	Food-biomass baseline, excl. interest/contingency	EU	hypothetical	9.6 EUR/kg DW	11.36	DW	Food-biomass tubular PBR baseline; excludes interest/contingency.	
Context/sensitivity	2021	Tubular PBR	Food-biomass boundary case, incl. interest/contingency	EU	hypothetical	12.43 EUR/kg DW	14.70	DW	Same model with interest and contingency included.	
Primary evidence	2021	Flat-panel PBR	100 ha GCC range	GCC	hypothetical	3.0–3.2 EUR/kg biomass	3.549–3.786	biomass	Primary GCC 100-ha range; shown as source-reported range.	[111]
Primary evidence	2021	Horizontal tubular PBR	100 ha GCC range	GCC	hypothetical	4.3–4.9 EUR/kg biomass	5.087–5.797	biomass	Primary GCC 100-ha range; shown as source-reported range.	
Primary evidence	2021	Open raceway pond	100 ha GCC range	GCC	hypothetical	2.9–3.5 EUR/kg biomass	3.431–4.141	biomass	Primary GCC 100-ha range; shown as source-reported range.	
Primary evidence	2021	Vertical stacked tubular PBR	100 ha GCC range	GCC	hypothetical	4.1–4.6 EUR/kg biomass	4.85–5.442	biomass	Primary GCC 100-ha range; shown as source-reported range.	
Primary evidence	2016	GWP-II flat-panel PBR	1 ha Tuscany, with cooling	Italy	hypothetical or demo informed	12.4 EUR/kg DW	13.73	DW	Primary 1-ha GWP-II base case to wet paste, DW basis.	[102]
Context/sensitivity	2016	GWP-II flat-panel PBR	1 ha Tunisia, no cooling	Tunisia	hypothetical location	5.4 EUR/kg DW	5.978	DW	Scale/location/no-cooling scenario; context.	
Context/sensitivity	2016	GWP-II flat-panel PBR	1 ha Tunisia, with cooling	Tunisia	hypothetical location	6.2 EUR/kg DW	6.863	DW	Scale/location/no-cooling scenario; context.	
Context/sensitivity	2016	GWP-II flat-panel PBR	1 ha Tuscany, thermotolerant/no cooling	Italy	hypothetical	11.1 EUR/kg DW	12.29	DW	Scale/location/no-cooling scenario; context.	
Context/sensitivity	2016	GWP-II flat-panel PBR	100 ha Tunisia, scaled	Tunisia	hypothetical scaled location	3.2 EUR/kg DW	3.542	DW	Scale/location/no-cooling scenario; context.	
Context/sensitivity	2016	GWP-II flat-panel PBR	100 ha Tunisia, scaled + no cooling	Tunisia	hypothetical scaled location	2.6 EUR/kg DW	2.878	DW	Scale/location/no-cooling scenario; context.	
Context/sensitivity	2016	GWP-II flat-panel PBR	100 ha Tuscany, scaled	Italy	hypothetical scaled	5.1 EUR/kg DW	5.646	DW	Scale/location/no-cooling scenario; context.	
Context/sensitivity	2016	GWP-II flat-panel PBR	100 ha Tuscany, scaled + no cooling	Italy	hypothetical scaled	4.1 EUR/kg DW	4.539	DW	Scale/location/no-cooling scenario; context.	
Primary evidence	2025	Open raceway pond	High-lipid CAP feedstock case	US	hypothetical	691 $/ton AFDW	0.761	AFDW	Future modelled NREL CAP feedstock/farm case.	[31]
Primary evidence	2025	Open raceway pond	High-protein CAP feedstock case	US	hypothetical	688 $/ton AFDW	0.758	AFDW	Future modelled NREL CAP feedstock/farm case.	
