# Supplementary material for: From Microalgal Biomass to Products: Downstream Processing Technology Gaps and the Road to Commercial Diversification
Source: Microorganisms. 2026 Jun 24;14(7):1393. doi: 10.3390/microorganisms14071393 (PMC13414295; doi:10.3390/microorganisms14071393)
Supplement: Supplementary file 1 [file microorganisms-14-01393-s001.zip › microorganisms-4336140-supplementary S1.pdf]

Scopus Pipeline

Reference Manual

Bibliometric Analysis of Microalgae Research 1995–2024

A keyword-based, rule-driven classification pipeline
for Scopus journal article metadata (title, abstract, keywords)

Scripts covered:

`Scopus_downloader_nokey.py`
`wp1_scopus_common.py`
`wp1_scopus_01_build_master.py`
`wp1_scopus_02_classify_applications.py`
`wp1_scopus_03_build_food_subtopics.py`
`wp1_scopus_04_make_triptych.py`
`wp1_scopus_04b_make_food_overlap_figure.py`
`wp1_scopus_05_export_supplement.py`
`run_wp1_scopus_triptych.py`

1 Overview and Design

This pipeline performs a complete, reproducible bibliometric analysis of peer-reviewed journal publications on microalgae, covering the period 1995–2024. The input is Scopus metadata - specifically title, author-supplied keywords, and abstract text. No full-text retrieval is used. This is an intentional and methodologically sound decision: Scopus provides these three fields comprehensively for all journal articles, and they are fully sufficient for keyword-based thematic classification at corpus scale (N > 40,000 publications).

1.1 Comparison with pybliometrics

The WP1 pipeline deliberately does not use pybliometrics for the following reasons, while remaining methodologically equivalent in all core bibliometric operations:

Aspect	pybliometrics	WP1 Custom Pipeline
API access	Abstracted via Python classes	Direct REST calls (requests library)
Pagination	Handled internally	Custom: window → cursor → facet-partition (3-strategy cascade)
5,000-result limit	Abstracted away (may silently truncate)	Explicitly handled with cursor + recursive facet partitioning
Resume capability	Not built in	Full resume: processed EIDs tracked in seen_eids set
Abstract retrieval	Via AbstractRetrieval class	Direct Abstract Retrieval API (META_ABS view per EID)
Text normalisation	Not provided	norm_text(): Unicode NFKD, lowercase, non-alphanumeric stripping
Thematic classification	Not provided	Full 9-cluster applied + 9-cluster non-applied taxonomy
QC flags	Not provided	5 QC flags per record + review priority
Reproducibility	Depends on local cache	Fully deterministic: locked settings, _validate_outputs() check
Output	Python objects	Structured CSV/GZ + JSON audit trail + Excel supplement tables

> *ℹ Both approaches query the same Scopus REST endpoints and retrieve the same fields (EID, DOI, title, coverDate, publicationName, aggregationType, subtype, authkeywords, abstract). The WP1 pipeline adds corpus-scale robustness and a complete downstream classification layer that pybliometrics does not provide.*

1.2 Pipeline Architecture

The pipeline consists of six sequential steps, each producing structured output files consumed by the next step. A single locked runner script (run_wp1_scopus_triptych.py) orchestrates all steps and performs post-hoc validation.

#	Filter / Schritt	Bedingung / Detail
Ph. 0	**Scopus_downloader_nokey.py**	Downloads raw metadata via Scopus Search + Abstract Retrieval API → JSONL.GZ
St. 1	**wp1_scopus_01_build_master.py**	Filters raw dump → peer-journal master CSV
St. 2	**wp1_scopus_02_classify_applications.py**	Classifies every record → applied/non-applied + cluster labels
St. 3	**wp1_scopus_03_build_food_subtopics.py**	Extracts food subset → food subtopic classification
St. 4a	**wp1_scopus_04_make_triptych.py**	Renders Figure S1 (3-panel triptych, PDF + PNG)
St. 4b	**wp1_scopus_04b_make_food_overlap_figure.py**	Renders Figure S2 (food overlap figure, PDF + PNG)
St. 5	**wp1_scopus_05_export_supplement.py**	Exports 12 supplement tables (CSV + Excel)
Val.	**run_wp1_scopus_triptych.py _validate_outputs()**	Checks 25 output files + setting consistency + cross-count validation

2 Phase 0 - Data Acquisition (Scopus_downloader_nokey.py)

This script handles all communication with the Elsevier Scopus REST API. It operates in five configurable modes (MODE variable) and produces two compressed JSONL files that feed the downstream pipeline.

2.1 Search Query

The Scopus Search API is queried with a fixed TITLE-ABS-KEY expression, meaning the term must appear in the title, abstract, or author keywords of the record. The query is applied year by year (AND PUBYEAR = {year}) for 1995–2024:

```
TITLE-ABS-KEY(
  microalgae OR "micro-algae" OR microalgal OR microalga OR microphyte OR
  chlorella OR nannochloropsis OR dunaliella OR haematococcus OR
  scenedesmus OR tetraselmis OR arthrospira OR spirulina
)
```

The 13 search terms cover the most important genus names and common synonyms for microalgae. The TITLE-ABS-KEY field operator ensures broad but targeted retrieval.

2.2 Pagination Strategy

The Scopus Search API enforces a hard limit of 5,000 results per query. For microalgae research after 2010, many years exceed this threshold. The downloader automatically selects one of three strategies per year:

#	Filter / Schritt	Bedingung / Detail
**S1**	**Standard window pagination**	≤ 5,000 results: iterates start parameter in increments of 25 (SEARCH_PAGE_SIZE) until all records retrieved
**S2**	**Cursor-based pagination**	> 5,000 results + institutional cursor access: uses @cursor token from each response to page without limit
**S3**	**Recursive facet partitioning**	> 5,000 results + no cursor: function build_leaf_queries() / _recursive_partition() splits the year query by facets in order: SRCTYPE → LANGUAGE → DOCTYPE → OPENACCESS → EXACTSRCTITLE. Recursion continues until each leaf partition has ≤ 5,000 results or max depth (MAX_SPLIT_DEPTH = 8) is reached

ℹ Strategy selection is automatic and transparent. The function probe_cursor_access() and probe_facets_access() test available API capabilities at runtime. Oversized years that cannot be fully retrieved (neither cursor nor facets available) are logged to oversized_years_not_retrieved.csv.

2.3 HTTP Robustness (_robust_get)

All API calls are wrapped in _robust_get(), which implements exponential backoff for transient failures:

- HTTP 429 (Rate Limit), 500, 502, 503, 504 → retry with backoff
- Initial wait: 2.0 s, multiplied by 1.7 each attempt, capped at 120 s plus random jitter (0–1 s)
- Maximum 10 retries per request
- Retry-After header respected when present

2.4 Two-Phase Download

Phase	Mode	API Endpoint	View	Output File
1 Minimal dump	dump	https://api.elsevier.com/content/search/scopus	STANDARD	scopus_search_dump_minimal.jsonl.gz
2 Abstract enrichment	enrich_abstracts	https://api.elsevier.com/content/abstract/eid/{eid}	META_ABS	scopus_search_dump_with_abstracts.jsonl.gz

Phase 2 (enrich_with_abstracts) processes the minimal dump record by record, fetches the full abstract via the Abstract Retrieval API, and writes the enriched record to the output file. The function is resume-capable: already-processed EIDs are tracked in processed_eids set so that interrupted runs can continue without re-fetching.

When both files are present, build_master() (Step 1) reads the enriched dump first. Deduplication ensures that for any record appearing in both files, the enriched version (with abstract) wins.

3 Step 1 - Building the Peer-Journal Master (wp1_scopus_01_build_master.py)

This step reads the raw JSONL.GZ dump(s), applies a sequential filter cascade, constructs text representations, and writes a compressed master CSV. All counts are tracked for the audit trail.

3.1 Filter Cascade

Each record passes through seven filters in strict order. A record failing any filter is discarded and counted. The audit table audit_corpus_flow_step01.csv records the count after each filter:

#	Filter / Schritt	Bedingung / Detail
F1	**Valid JSON**	Malformed JSON lines are skipped via try/except json.JSONDecodeError
F2	**Year extraction**	parse_year_from_cover_date() applies regex ^(\d{4}) to coverDate (format YYYY-MM-DD). Fallback: search_year_bin field. Records with year = 0 are discarded
F3	**Deduplication**	Dedup key hierarchy: (1) EID, (2) DOI lowercased and stripped, (3) norm_text(title) + '|' + year. Key stored in seen_eids set; duplicates discarded. First-seen enriched record always wins
F4	**Year window**	Only records with 1995 ≤ year ≤ 2024 retained
F5	**Aggregation type**	aggregationType.strip().lower() == 'journal'. Excludes book chapters, conference papers, book series, trade publications
F6	**Document subtype**	subtype.strip().lower() in {'ar', 're'}. Only Articles (ar) and Reviews (re). Excludes editorials, letters, notes, errata, conference abstracts
F7	**Corpus Boundary Filter**	passes_light_corpus_boundary_filter(): RETAIN if has_direct_microalgae_anchor(text_core) OR is_whitelisted_algal_journal(publicationName). Logic is OR: either condition is sufficient

3.2 Corpus Boundary Filter - Complete Term Lists

3.2.1 Direct Microalgae Anchor Terms (28 terms, regex match in text_core)

These terms are matched case-insensitively via DIRECT_MICROALGAE_RE compiled regex:

Term	Term	Term	Term
microalgae	microalgal	micro alga	micro algae
micro-alga	micro-algae	alga	algal
algae	cyanobacteria	cyanobacterial	chlorella

spirulina	arthrospira	nannochloropsis	haematococcus
dunaliella	scenedesmus	tetraselmis	chlamydomonas
isochrysis	porphyridium	phaeodactylum	nitzschia
pavlova	botryococcus	synechococcus	synechocystis

3.2.2 Whitelisted Algal Journals (9 journals)

Records from these journals are retained regardless of whether microalgae terms appear in the text (ALGAL_JOURNAL_WHITELIST_RE, case-insensitive match on publicationName):

- Algal Research
- Journal of Applied Phycology
- Journal of Phycology
- Phycologia
- Phycological Research
- European Journal of Phycology
- Algological Studies
- Harmful Algae
- Cryptogamie Algologie

3.3 Text Representation Construction

Three text fields are built for each retained record, all processed through norm_text():

> *ℹ norm_text(): Unicode NFKD normalisation → lowercase → replace all non-[a-z0-9] characters with space → collapse multiple spaces → strip*

Field	Composition	Used for
text_core	norm_text(title + ' ' + authkeywords + ' ' + abstract_text)	Primary classification (Steps 2, 3)
text_source_augmented	text_core + ' ' + norm_text(publicationName + ' ' + subtypeDescription)	QC comparison: does adding journal name change classification?
text_rich	Alias for text_source_augmented	Legacy compatibility

3.4 Output

Records are written to wp1_scopus_master.csv.gz in batches of 20,000 rows to manage memory. Output columns include all Scopus metadata fields plus text_core, text_source_augmented, text_rich, boundary filter flags (boundary_has_direct_micro_anchor, boundary_whitelisted_journal), and all original API fields (issn, eIssn, source_id, citedby_count, openaccess).

4 Step 2 - Thematic Classification (wp1_scopus_02_classify_applications.py)

This is the analytical core of the pipeline. The function classify_application_record() is applied to every record and produces approximately 60 new columns covering cluster membership, scoring, QC flags, non-applied classification, and display labels.

4.1 Scoring System

For every record, each of the 9 Applied clusters receives a numerical score computed by _score_rulebook():

```
Score = W_HARD_ANCHOR × hard_anchor_hits + W_ANCHOR × anchor_hits + W_SUPPORT ×
support_hits

Constants: W_HARD_ANCHOR = 6 W_ANCHOR = 2 W_SUPPORT = 1

Phrase weighting:   multi-word phrases (e.g. 'food ingredient') count 2×;
                    single-word terms count 1× (via weighted_hit_count())
```

4.2 Three Term Levels

Each cluster has three tiers of evidence terms with different scoring weights:

- Hard Anchors (×6 per weighted hit): Very specific, high-confidence terms that unambiguously signal the cluster. A single hard anchor match scores 6–12 points and triggers qualification regardless of other scores.
- Anchors (×2): Strong but not absolute signal terms. Require supporting context to qualify a cluster on their own.
- Supports (×1): Weaker contextual terms. Alone they are insufficient; in combination with anchors they confirm cluster membership.

> *ℹ Hard Anchors are defined in a separate HARD_DOMAIN_ANCHORS dict in wp1_scopus_common.py, while regular Anchors and Supports live in CLUSTERS. The _score_rulebook() function takes both structures as input and applies the ×6 weight to HARD_DOMAIN_ANCHORS terms*

4.3 Qualification Logic

A cluster qualifies (becomes a member of the record's cluster set) if ANY of the following conditions is met:

- Q1: hard_hit ≥ 1 (at least one hard anchor term matched)
- Q2: anchor_hit ≥ 1 AND support_hit ≥ 1 (anchor + supporting context)
- Q3: anchor_hit ≥ 2 (two or more anchor matches, no support needed)
- Q4: score ≥ 6 (MIN_SCORE_BYPASS; any combination reaching score 6)

> *ℹ Qualification is evaluated per cluster independently. A single record can qualify for multiple clusters simultaneously - this produces multi-membership (cluster_multi field).*

4.4 Applied Cluster Taxonomy - Complete Keyword Tables

The following tables list all terms for each of the 9 Applied clusters. Red = Hard Anchors (weight ×6), Orange = Anchors (weight ×2, unique to this tier), Blue = Supports (weight ×1).

Food and food ingredients [Precedence: 50]

Hard Anchors (×6)	Anchors (×2)	Supports (×1)
food ingredient	plant based	food
food product	plant-based	ingredient
novel food		formulation
edible		incorporation
bakery		sensory
bread		taste
pasta		flavour
noodle		flavor
beverage		aroma
drink		odor
dairy		odour
yogurt		texture
cheese		textur
meat analog		emulsion
meat analogue		foaming
consumer acceptance		gel
		gelation
		rheolog
		viscos
		solubility
		digestibility
		protein isolate
		protein ingredient

Nutraceuticals / supplements [Precedence: 55]

Hard Anchors (×6)	Anchors (×2)	Supports (×1)
nutraceutical	supplement	bioactive

dietary supplement		antioxidant
functional ingredient		omega 3
omega 3 supplement		epa
		dha
		astaxanthin
		lutein
		zeaxanthin
		phycocyanin
		vitamin

Pharmaceuticals / healthcare [Precedence: 75]

Hard Anchors (×6)	Anchors (×2)	Supports (×1)
pharmaceutical	drug	medical
drug delivery		cancer
therapeutic		antiviral
clinical		antibacterial
clinical trial		antimicrobial
anticancer		immun
cutaneous		toxicity
protothecosis		inflammation
		disease

Feed / aquaculture [Precedence: 80]

Hard Anchors (×6)	Anchors (×2)	Supports (×1)
aquaculture	feed	fish
feed additive		larvae
broiler		hatchery
salmon		broodstock
shrimp		tilapia
broodstock		
livestock		
poultry		
tilapia		

feed supplement		
live feed		
larval diet		

Biofuels / bioenergy [Precedence: 85]

Hard Anchors (×6)	Anchors (×2)	Supports (×1)
biodiesel		hydrogen production
bioethanol		lipid
biogas		anaerobic digestion
biofuel		hydrothermal
bioenergy		
pyrolysis		
hydrothermal liquefaction		
htl		
gasification		
transesterification		
anaerobic digestion		

Wastewater / environmental remediation [Precedence: 90]

Hard Anchors (×6)	Anchors (×2)	Supports (×1)
wastewater		heavy metal
waste water		dye removal
phycoremediation		micropollutant
bioremediation		co2 capture
water treatment		carbon capture
nutrient removal		flue gas
nitrogen removal		
phosphorus removal		

Cosmetics / pigments / colorants [Precedence: 70]

Hard Anchors (×6)	Anchors (×2)	Supports (×1)
cosmetic	colorant	phycocyanin
cosmeceutical	colourant	astaxanthin

**skincare**	**pigment**	carotenoid
**skin care**		lutein
**sunscreen**		chlorophyll
**personal care**		uv
		anti aging
		anti-aging

Agriculture / biostimulants / fertilizers [Precedence: 60]

Hard Anchors (×6)	Anchors (×2)	Supports (×1)
**biofertilizer**		plant growth
**fertilizer**		crop
**biostimulant**		soil
**bio-stimulant**		rhizosphere
**foliar spray**		yield improvement

Biomaterials / bioplastics / circular economy [Precedence: 65]

Hard Anchors (×6)	Anchors (×2)	Supports (×1)
**bioplastic**	**biochar**	material
**biolubricant**	**circular economy**	graphene
**lubricant**		nanocellulose
**tribology**		platform chemical
**tribological**		waste valorization
**composite**		valorization
**hydrogel**		
**resin**		
**polymer**		

4.5 Cluster Ranking and Primary Assignment

When multiple clusters qualify, they are ranked by the following composite key (descending):

```
ranked = sorted(qualified_clusters, key=lambda k: (hard_hit[k], score[k],
CLUSTER_PRECEDENCE[k]), reverse=True)
```

The top-ranked cluster becomes cluster_primary. All qualified clusters are stored in cluster_multi (semicolon-separated).

Precedence values serve as a tiebreaker when hard_hit and score are equal:

Cluster	Precedence
Wastewater / environmental remediation	90
Biofuels / bioenergy	85
Feed / aquaculture	80
Pharmaceuticals / healthcare	75
Cosmetics / pigments / colorants	70
Biomaterials / bioplastics / circular economy	65
Agriculture / biostimulants / fertilizers	60
Nutraceuticals / supplements	55
Food and food ingredients	50

4.6 Special Rules for Food Cluster

The Food cluster has two additional constraints applied after the standard qualification check:

4.6.1 Food Specificity Gate

Even if the score threshold is met, the Food cluster only qualifies if at least one of the following food-specific terms is present in text_core:

Food-specific gate terms (any one sufficient)
formulation, sensory, taste, flavor, flavour, texture, digestibility, beverage, bakery, bread, pasta, yogurt, cheese, meat analog, meat analogue, food product, food ingredient

4.6.2 Food Blocker List

If any of the following terms are present in text_core, the Food cluster is immediately disqualified (CLUSTER_BLOCKERS["Food and food ingredients"]):

Blocker terms for Food cluster (presence of any → Food disqualified)
biodiesel, bioethanol, biogas, biofuel, bioenergy, pyrolysis, hydrothermal liquefaction, htl, gasification, transesterification, anaerobic digestion, wastewater, waste water, phycoremediation, water treatment, nutrient removal, nitrogen removal, phosphorus removal, aquaculture, feed additive, broiler, salmon, shrimp, livestock, pharmaceutical, drug delivery, clinical, anticancer, cosmetic, skincare, sunscreen, bioplastic, biolubricant, polymer, hydrogel, engine, bioremediation, drug delivery, anticancer

4.6.3 Nutraceuticals Blocker List

Blocker terms for Nutraceuticals / supplements
drug delivery, clinical trial, anticancer

4.7 Ambiguity Detection and QC Flags

After classification, five QC flags are set per record to support manual review and audit:

#	Filter / Schritt	Bedingung / Detail
QC1	**qc_ambiguous**	applied=True AND (primary_score < 4 OR primary_margin < 3). Indicates the classification is uncertain - the primary cluster's score is low or the margin over the runner-up is small
QC2	**qc_generic_primary_only**	All matched terms for the primary cluster are generic (e.g. only 'food' for Food cluster) AND no hard anchor was hit. Indicates weak evidence.
QC3	**qc_large_multi_membership**	3 or more clusters qualified simultaneously (QC_MULTI_THRESHOLD = 3). Indicates a highly interdisciplinary record.
QC4	**qc_food_blocker_cooccurrence**	Text contains Food-related terms AND Food-blocker terms simultaneously. Flags records where food language and process/pharma language co-occur.
QC5	**qc_source_title_changed**	Classification changes when text_source_augmented (includes journal name) is used instead of text_core. Indicates the journal name is influencing the result.

Based on these flags, each record receives a review_priority:

- high: source_title_changed = True OR food_blocker_cooccurrence = True OR (ambiguous AND primary_margin ≤ 1)
- medium: any QC flag set
- low: no QC flags

4.8 Clean Primary Assignment (cluster_primary_clean)

cluster_primary_clean is only populated when:

- applied_flag = True (at least one cluster qualified)
- qc_ambiguous = False (primary score ≥ 4 AND margin ≥ 3)

This field represents unambiguous, high-confidence cluster assignments. It is used as the basis for Panel B of the triptych figure and for Food subset selection in Step 3.

4.9 Ambiguity Bucket Decomposition

Records where applied=True but cluster_primary_clean is empty (i.e. ambiguous applied records) are assigned to one of five Ambiguity Buckets via nominate_applied_other_bucket():

Family code	Plot label	Constituent clusters
resource_process	Process	Biofuels / bioenergy, Wastewater / environmental remediation, Biomaterials / bioplastics / circular economy

bioactivity_health	Bioactive	Pharmaceuticals / healthcare, Nutraceuticals / supplements, Cosmetics / pigments / colorants
feed_agri	Feed/agri	Feed / aquaculture, Agriculture / biostimulants / fertilizers
food_adjacent	Residual	Food and food ingredients
cross_family_residual	Residual	Fallback: multi-family ambiguity not resolved

4.10 Non-Applied Classification

Records where no Applied cluster qualifies (applied_flag = False) are independently classified by _classify_nonapplied_text_internal() using a parallel 9-cluster Non-Applied taxonomy. The scoring mechanics are identical (same W_HARD_ANCHOR / W_ANCHOR / W_SUPPORT constants, same qualification logic). A fallback mechanism (NONAPPLIED_FALLBACK_TERMS) handles records that fail standard qualification but show weak evidence for a non-applied mode.

Non-Applied Cluster Keyword Tables

Biochemistry / composition [Precedence: 50]

Hard Anchors (×6)	Anchors (×2)	Supports (×1)
**biochemical composition**	**pigment profile**	metabol
**proximate composition**	**metabolite profiling**	metabolite
**fatty acid profile**	**biochemical characterization**	composition
**amino acid profile**	**enzyme activity**	biochemistry
**biosynthetic pathway**	**enzymatic activity**	biochemical
**cell wall composition**		fatty acid
		lipid
		protein
		polysacchar
		carbohydrate
		pigment
		chlorophyll
		carotenoid
		phycocyanin
		amino acid
		proximate
		biosynth
		biosynthetic
		biosynthesis

		pathway
		enzyme
		enzymatic
		dehydrogenase
		kinase
		phosphatase
		sterol
		cell wall
		sugar
		extract
		metabolic
		hydrolysate
		hydrolyzate

Ecology / environmental dynamics [Precedence: 65]

Hard Anchors (×6)	Anchors (×2)	Supports (×1)
**phytoplankton community**	**community composition**	ecolog
**harmful algal bloom**	**algal bloom**	ecological
**seasonal succession**	**species distribution**	phytoplankton
**marine ecosystem**	**freshwater ecosystem**	community
**population dynamics**	**trophic interaction**	bloom
**feeding biology**	**grazing pressure**	lake
		river
		marine
		ocean
		coastal
		freshwater
		estuar
		benthic
		reservoir
		trophic
		grazing
		feeding
		population

		interaction
		interactions
		shore
		periphyton
		epibiont
		rotifer
		daphnia
		copepod
		clam
		parrotfish
		gastropod
		resource use
		food density
		selectivity

Cultivation / reactor / media optimization [Precedence: 70]

Hard Anchors (×6)	Anchors (×2)	Supports (×1)
**photobioreactor**	**continuous culture**	cultivation
**culture conditions**	**batch culture**	culture medium
**cultivation conditions**	**reactor design**	medium
**media optimization**	**heterotrophic culture**	reactor
**growth kinetics**		bioreactor
**high cell density culture**		photobioreactor
		light intensity
		nitrogen limitation
		co2 supplementation
		salinity
		temperature
		optimization
		biomass productivity
		cell density
		heterotrophic
		mixotrophic
		autotrophic

		growth rate
		culture
		cultures
		growing
		co2 concentration
		carbon fixation rate
		nutrient limited

Toxicology / pollutant response [Precedence: 60]

Hard Anchors (×6)	Anchors (×2)	Supports (×1)
**ecotoxicity**	**pollutant stress**	tox
**toxicity assessment**	**phytotoxicity**	toxicity
**nanoparticle toxicity**		pollut
**metal stress**		heavy metal
**inhibitory effect**		cadmium
		lead
		arsenic
		copper
		zinc
		herbicide
		pesticide
		nanoparticle
		exposure
		algicide
		phthalate
		pentachlorophenol
		monocrotophos
		inhibitive effect

Analytical methods / characterization [Precedence: 55]

Hard Anchors (×6)	Anchors (×2)	Supports (×1)
**method development**	**chromatographic analysis**	method
**method validation**	**microscopy analysis**	methods
**analytical method**		analysis

**spectroscopic characterization**		analytical
**rapid differentiation test**		characterization
		spectros
		chromat
		microscop
		imaging
		assay
		profiling
		quantification
		test
		differentiation
		stability

Physiology / photosynthesis / stress [Precedence: 75]

Hard Anchors (×6)	Anchors (×2)	Supports (×1)
**photosynthetic efficiency**	**photoinhibition**	photosynth
**chlorophyll fluorescence**		physiology
**photophysiology**		photophysiology
**stress physiology**		fluorescence
**inorganic carbon uptake**		chlorophyll fluorescence
**osmotic shock**		light stress
		nitrogen stress
		salt stress
		stress
		acclimation
		photoinhibition
		inorganic carbon
		carbon uptake
		redox
		plasma membrane
		homeostasis
		carbon fixation
		uptake

Omics / molecular biology [Precedence: 80]

Hard Anchors (×6)	Anchors (×2)	Supports (×1)
transcriptome	genomics	genome
proteome		transcript
metabolome		proteom
genome sequencing		metabolom
rna seq		gene expression
ribonucleotide reductase		molecular
		sequencing
		omics
		rna
		dna
		deoxyribonucleotide
		translation
		transcription

Taxonomy / biodiversity [Precedence: 85]

Hard Anchors (×6)	Anchors (×2)	Supports (×1)
taxonomy	phylogeny	phylog
phylogenetic analysis	species identification	taxonom
barcoding		barcode
biodiversity		biodiversity
new species		systematics
fossil record		genus
		species complex
		fossil
		cretaceous
		flora
		occurrence
		chlorophyta

Strain engineering / mutagenesis [Precedence: 90]

Hard Anchors (×6)	Anchors (×2)	Supports (×1)
genetic engineering	transgenic	mutant

**metabolic engineering**		mutation
**mutagenesis**		transformation
**transformation**		engineered strain
**gene editing**		overexpression
**crispr**		knockout
		crispr
		editing
		recombinant
		strain improvement
		selection strategy

4.11 Crosscut Tags

Nine crosscut tags are evaluated for every record independently of the applied/non-applied classification. They capture cross-cutting methodological or thematic properties that span multiple clusters:

Tag name	Keywords (all must appear as substrings in norm_text(text_core))
biorefinery_context	biorefinery, valorization, valorisation, cascade use, integrated process, integrated bioprocess
integrated_processing	integrated processing, integrated process, integrated biorefinery, cascade
downstream_processing	downstream, filtration, membrane, ultrafiltration, microfiltration, diafiltration, centrifug, floccul, drying, spray drying, freeze drying
extraction_fractionation	extraction, fractionation, purification, isolate, separation
protein_focus	protein, peptide, amino acid
pigment_focus	pigment, phycocyanin, astaxanthin, carotenoid, chlorophyll, lutein
lipid_focus	lipid, fatty acid, triacylglycerol, omega 3, epa, dha
waste_to_product	waste valorization, waste valorisation, by product, by-product, co product, co-product
cultivation_coupling	photobioreactor, cultivation, culture condition, media optimization, light intensity, co2 supplementation

5 Step 3 - Food Subtopic Classification (wp1_scopus_03_build_food_subtopics.py)

This step extracts the food-specific subset of the classified master and applies a three-level classification to assign each food publication to a detailed subtopic.

5.1 Food Subset Selection

Subset mode: primary_clean (locked). Only records where cluster_primary_clean == 'Food and food ingredients' are included. This means the record must be: applied_flag = True AND qc_ambiguous = False AND primary cluster = Food.

Classification text used: text_core (food_text_mode = 'core'). The journal name is not used for food subtopic classification.

5.2 Level 1 - Base Subtopic Assignment (pick_best)

The function pick_best(text_core, FOOD_SUBTOPICS, min_hits=1) computes a score for each of the 8 food subtopics by counting keyword matches (multi-word phrases score 2×, single words score 1×). The subtopic with the highest score (minimum 1 hit) is assigned. Ties go to the first-listed subtopic. Records with zero hits receive 'Other'.

Subtopic	All classification keywords
Protein / nutrition	protein, amino acid, nutrition, digest, bioaccess, bioavail, protein quality
Extraction / fractionation / biorefinery	extraction, fractionation, isolate, purification, biorefinery, cascade, integrated
Processing / downstream / drying	filtration, membrane, ultrafiltration, microfiltration, diafiltration, centrifug, floccul, drying, spray drying, freeze drying, lyophil, pasteur, steriliz, heat treatment
Functionality / techno-functional	emulsion, foaming, gel, gelation, rheolog, viscos, solubility, stability, water holding
Sensory / flavor / odor / color	sensory, taste, flavour, flavor, aroma, odor, odour, color, colour
Formulation / product development	formulation, incorporation, food product, prototype, recipe, bakery, bread, pasta, noodle, dairy, yogurt, cheese, meat analog, plant based, plant-based, beverage
Texturization / structuring	textur, extrusion, shear cell, fibrous, structur, 3d print, 3 d print
Safety / digestibility / regulation	safety, toxin, heavy metal, contamin, allergen, regulation, novel food regulation, digestibility

5.3 Level 2a - Reassignment from 'Other' back to Main Subtopics (FOOD_OTHER_REASSIGN)

Records that received 'Other' in Level 1 are first tested against a targeted reassignment dictionary (FOOD_OTHER_REASSIGN). If any terms match, the record is reassigned to the corresponding main subtopic:

Target subtopic	Reassignment trigger terms
Protein / nutrition	prebiotic, healthy lipids, fatty acid, edible lipids, single cell oil, sport supplement, food additive, astaxanthin accumulation
Safety / digestibility / regulation	novel food, legal framework, regulatory, nitrate, nitrite, iodine concentrations, commercial edible algae
Formulation / product development	coating, packaging, post harvest, postharvest, mango, tomato, modified atmosphere
Processing / downstream / drying	cryopreservation

5.4 Level 2b - Crossover Bucket Assignment (FOOD_OTHER_BUCKET_RULES)

Remaining 'Other' records are tested against 6 data-driven crossover buckets (FOOD_OTHER_BUCKET_RULES). These capture records that were classified as Food but contain language from adjacent research domains:

Crossover bucket	Trigger keywords
Food-other: water/ecology crossover	drinking water, water quality, reservoir, river, lake, estuary, chlorination, trihalomethane, haloacetic, phytoplankton, community, ecosystem, cladocer, zooplankton, benthic, grazing, food chain, trophic, water tox, water types, arsenic mobilisation, organic matter, cyanobacterial fluorescence, species diversity, brachionus, sardinella, water temperature
Food-other: health/bioactivity crossover	rats, mice, mouse, colitis, nephrotox, carcinogenesis, cytokine, lead exposed, anti inflammatory, biofilm, urinary catheter, liver, blood mononuclear, ulcerative, oxidative stress, infected with, in vitro, chemopreventive, therapeutic, mental fatigue, healthy volunteers, haematological, hemato toxicity, fluoride, aluminum, microbial health industry
Food-other: process/energy/materials crossover	green hydrocarbons, jet fuel, diesel, catalytic deoxygenation, nanoparticle, green synthesis, effluent, photobioreactor, electric stimulation, desalination brine, process design, simulation, metal nanoparticles, hydrocarbons, electric arc furnace, steel slag, microwave absorption, polyethylene, polypropylene, biosorption, cr iii
Food-other: organism/strain characterization → platform crossover	genome sequence, whole genome shotgun, edible cyanobacterium, characteristics of edible cyanobacteria, strain paraca, pigment biosynthesis
Food-other: cultivation/biomass crossover → platform crossover	biomass productivity, biomass concentration, daily biomass, grown in, continuous bioreactor, dual growth limitation, estimating microalgae
Food-other: feed/livestock crossover → platform crossover	laying hens, egg production, rotifer, rumen, hay, broiler, sea urchin

> *ℹ The last three buckets (organism/strain, cultivation/biomass, feed/livestock) are collapsed to 'Food-other: platform crossover' for plotting purposes. Records with no bucket match remain as 'Residual food-other'.*

5.5 Panel C Threshold Collapsing

For the treemap visualisation (Panel C), subtopics with less than 2.0% share of total food publications (PANEL_C_OTHER_THRESHOLD_PCT) are collapsed to 'Other (<2%)'. Detailed assignments remain in the master file and supplement tables.

6 Steps 4a & 4b - Figure Generation

6.1 Figure S1: Triptych (wp1_scopus_04_make_triptych.py)

Panel	Data source	Visualisation	Key parameters
A - Field structure	display_primary_class from classified master, all records	Stacked area chart: all 9 Non-Applied + 9 Applied clusters + Other + Residual non-applied. Black line separates Non-Applied from Applied.	No smoothing; raw annual counts
B - Applied focus	panelB_display_class_plot, applied_flag=True records only	Stacked area chart: 9 Applied clusters + 4 Ambiguity-Bucket plot labels, normalised to 100% per year. Second Y-axis: Biorefinery-context share (dashed line).	3-year centred rolling mean (smooth_series); BIOREF_RE regex for biorefinery line
C - Food topics	food_subtopic_plot from food subtopics master	Treemap: custom Squarify implementation. Area proportional to record count. Labels placed at up to 6 font sizes (7.2→5.3pt), both horizontal and rotated 90°.	Subtopics < 2% collapsed to 'Other (<2%)'

Biorefinery context regex (BIOREF_RE) - a record's applied_flag=True count contributes to the biorefinery share if the following regex matches text_core (case-insensitive):

BIOREF_RE matched patterns
bio(-)refiner(y/ies/ing), valor(i)(z/s)ation, valor(i)(z/s)e, co(-)(product(s)), by(-)(product(s)), cascade( )use, integrated (process / approach / bioprocess / biorefinery)

6.2 Figure S2: Food Overlap (wp1_scopus_04b_make_food_overlap_figure.py)

Population: all applied records where Food appears in cluster_multi OR cluster_primary OR cluster_primary_clean.

- Panel A (UpSet-style): Top 12 exact combination frequencies counted via Python Counter. Each combination is a tuple of all cluster memberships for that record.
- Panel B (Pairwise): For each of the 8 comparison clusters, the count and percentage of Food-linked records that also have that cluster in their cluster_multi membership is computed.

Comparison clusters: Feed / aquaculture, Nutraceuticals / supplements, Pharmaceuticals / healthcare, Cosmetics / pigments / colorants, Biofuels / bioenergy, Wastewater / environmental remediation, Biomaterials / bioplastics / circular economy, Agriculture / biostimulants / fertilizers.

7 Step 5 - Supplement Export and Pipeline Validation

7.1 Supplement Tables

File	Format	Contents
Table_S1_corpus_flow_scopus.csv	CSV	Step-by-step funnel: record count after each of the 7 filters in Step 1
Table_S2_application_rulebook.csv	CSV	All 9 Applied clusters with complete Hard Anchor / Anchor / Support term lists
Table_S2b_qc_framework_scopus.csv	CSV	QC flag definitions and threshold values
Table_S2c_crosscut_tag_rulebook_scopus.csv	CSV	All 9 crosscut tags with keyword lists
Table_S2d_nonapplied_rulebook_scopus.csv	CSV	All 9 Non-Applied clusters with complete term lists
Table_S2e_other_reason_scopus.csv	CSV	Breakdown of 'Other' reason codes (low_margin, source_title_sensitive, blocker_conflict, high_overlap, generic_only, unresolved)
Table_S2f_boundary_filter_scopus.csv	CSV	Boundary filter statistics: records retained by microalgae anchor vs. journal whitelist
Table_S2g_applied_other_bucket_rulebook_scopus.csv	CSV	Applied-Other Ambiguity Bucket definitions and family assignments
Table_S3_food_subtopic_rulebook.csv	CSV	Food subtopic keyword lists (Level 1, 8 subtopics)
Table_S3b_food_other_bucket_rulebook_scopus.csv	CSV	Food-Other crossover bucket keyword lists (Levels 2a and 2b)
Table_S4_underlying_panel_counts.xlsx	Excel	Panel A, B, C annual counts (formatted with headers)
Table_S5_residual_nonapplied_qc.xlsx	Excel	Residual non-applied records with QC flags

7.2 Post-Pipeline Validation (_validate_outputs)

After all pipeline steps complete, run_wp1_scopus_triptych.py calls _validate_outputs() which performs three independent checks:

#	Filter / Schritt	Bedingung / Detail
**V1**	**File completeness**	All 25 expected output files exist on disk (checked with Path.is_file())

**V2**	**Setting consistency**	food_selection_mode and food_text_mode in wp1_scopus_food_subset_metadata.json match PIPELINE_SETTINGS. panel_cluster_mode in wp1_scopus_panel_metadata.json matches PIPELINE_SETTINGS.
**V3**	**Cross-count integrity**	sum(applied_flag == True) in the classified master CSV must equal the sum of all values in wp1_scopus_panelB_counts.csv (excluding the year column). A mismatch indicates that classification and plotting data have diverged.

> *ℹ The pipeline raises RuntimeError on any validation failure, preventing silent downstream errors. This deterministic validation step is the equivalent of a unit test for the entire pipeline run.*

8 Common Module Reference (wp1_scopus_common.py)

All shared constants, taxonomies, scoring logic, and utility functions live in wp1_scopus_common.py. This centralises the complete analytical specification and ensures all pipeline steps use identical definitions.

8.1 Fixed Constants

Constant	Value	Description
YEAR_MIN / YEAR_MAX	1995 / 2024	Analysis window
W_HARD_ANCHOR	6	Scoring weight for hard anchor terms
W_ANCHOR	2	Scoring weight for anchor terms
W_SUPPORT	1	Scoring weight for support terms
MIN_SCORE_BYPASS	6	Score threshold for cluster qualification (Q4)
MIN_PRIMARY_SCORE	4	Minimum primary score for non-ambiguous assignment
MIN_PRIMARY_MARGIN	3	Minimum margin over runner-up for non-ambiguous assignment
QC_MULTI_THRESHOLD	3	Minimum cluster count for large_multi_membership flag
PANEL_C_OTHER_THRESHOLD_PCT	2.0	Food subtopics below this % share are collapsed to 'Other (<2%)'
SMOOTH_YEARS	3	Window width for Panel B rolling mean smoothing
DPI_PNG	900	Resolution for PNG figure exports
BOUNDARY_FILTER_ENABLED	True	Whether to apply corpus boundary filter in Step 1

8.2 Key Functions

Function	Purpose
norm_text(s)	Unicode NFKD normalisation → lowercase → replace non-[a-z0-9] with space → strip. Applied to all text before any matching.
build_text_core(title, keywords, abstract)	Assembles and normalises the primary classification text.
build_text_source_augmented(...)	Adds publicationName and subtypeDescription to text_core for QC comparison.
has_direct_microalgae_anchor(text)	Returns True if any of the 26 microalgae anchor terms match in norm_text(text) via regex.
is_whitelisted_algal_journal(pub_name)	Returns True if publicationName matches any of the 9 whitelisted journals.

passes_light_corpus_boundary_filter(text, pub_name)	Returns has_direct_microalgae_anchor(text) OR is_whitelisted_algal_journal(pub_name).
classify_application_record(text_core, text_aug)	Main classification function. Runs _classify_application_text_internal() on both text fields, _classify_nonapplied_text_internal() for non-applied mode, computes all QC flags, sets display classes. Returns dict with ~40 fields.
_score_rulebook(text, rulebook, ...)	Core scoring engine. For each cluster: counts hard anchor hits, anchor hits, support hits; computes weighted score; tests qualification; returns ranked results.
_classify_nonapplied_text_internal(text)	Runs _score_rulebook() on NONAPPLIED_MODES. Falls back to NONAPPLIED_FALLBACK_TERMS if standard qualification fails. Returns primary non-applied mode and QC fields.
nominate_applied_other_bucket(...)	Assigns ambiguous applied records to one of 5 ambiguity families based on which clusters qualify and their scores.
nominate_food_other_bucket(text)	Two-stage residual assignment: (1) FOOD_OTHER_REASSIGN → back to main subtopics; (2) FOOD_OTHER_BUCKET_RULES → crossover buckets.
pick_best(text, mapping, min_hits)	Finds the highest-scoring entry in a keyword→score mapping. Used for food subtopic assignment.
pivot_year_counts(df, year_col, cat_col)	Groups DataFrame by year and category column, pivots to year×category matrix, reindexes to full YEAR_RANGE.
smooth_series(y, w)	Centred rolling mean with window w (default 3 years). Used for Panel B.
counts_to_share(piv)	Normalises pivot table row-wise to percentages (sum = 100% per year).
squarify(sizes, x, y, dx, dy)	Custom implementation of the Squarify treemap algorithm. Minimises worst aspect ratio for Panel C.
export_simple_workbook(sheets, path)	Writes formatted Excel workbook via openpyxl with coloured header row and auto-column widths.
iter_jsonl_gz(path)	Memory-efficient line-by-line iterator over gzip-compressed JSONL files.
parse_year_from_cover_date(cover_date)	Extracts 4-digit year from Scopus coverDate string (YYYY-MM-DD or YYYY).
find_input_dumps()	Discovers JSONL.GZ input files; orders enriched before minimal for merge-mode deduplication.

9 Reproducibility and Methodological Transparency

The pipeline is designed for full reproducibility and transparent methodology documentation. Key design decisions that support this:

9.1 Determinism

- All scoring thresholds, term lists, and cluster definitions are fixed constants in wp1_scopus_common.py. No machine learning, no probabilistic models, no random initialisation.
- The only random element is jitter in HTTP backoff (random.uniform(0, 1.0)) which affects download timing only, not results.
- PIPELINE_SETTINGS dict in run_wp1_scopus_triptych.py locks food_selection_mode, food_text_mode, and panel_cluster_mode at runtime. Any deviation from these settings raises RuntimeError in _validate_outputs().

9.2 Audit Trail

Every pipeline step writes audit files documenting what happened:

- audit_corpus_flow_step01.csv: exact record counts at each of the 7 filters
- audit_build_master.json: full dump of counts, year range, boundary filter logic
- audit_boundary_filter_summary.csv: how many records were retained by each boundary condition
- audit_application_qc_summary.csv: counts for every QC flag and classification metric
- audit_application_cluster_counts.csv: distribution of cluster_primary values
- audit_other_reason_counts.csv: distribution of other_reason codes
- audit_applied_other_bucket_counts.csv: distribution of ambiguity bucket assignments
- wp1_scopus_food_subset_metadata.json: food subset selection parameters and counts
- wp1_scopus_panel_metadata.json: panel configuration and column mapping

9.3 Supplement Tables as Rulebook Documentation

Tables S2 through S3b in the supplement package reproduce the complete classification rulebook in tabular form - every term, every threshold, every rule - so that the classification can be independently verified, replicated, or extended by any researcher with access to a Scopus subscription.

9.4 Why Rule-Based Rather Than Machine Learning

A keyword-based rule system was chosen deliberately over machine learning classification for the following reasons:

- Full transparency: every classification decision can be traced to specific terms in the text and specific rules in the rulebook.
- No training data dependency: the classifier does not require labelled examples, which are difficult to obtain at scale for a niche research domain.

- Robustness across time: the taxonomy is defined by the research community's established terminology, which changes slowly. A machine learning classifier trained on data from one decade may not generalise to another.
- Ease of audit: reviewers can inspect any record's classification by examining its score__ columns, anchors__ columns, and blocker__ columns directly in the classified master CSV.
- Compatibility with supplement documentation: the complete rulebook can be published as supplementary tables (Table S2–S3b), satisfying journal requirements for methodological transparency.

10 Complete Output File Index

File	Directory	Format	Produced by
wp1_scopus_master.csv.gz	data/	CSV.GZ	Step 1
wp1_scopus_classified_master.csv.gz	data/	CSV.GZ	Step 2
wp1_scopus_panelB_counts.csv	data/	CSV	Step 4a
wp1_scopus_food_subtopics_master.csv.gz	data/	CSV.GZ	Step 3
wp1_scopus_food_subtopics_by_year.csv	data/	CSV	Step 3
wp1_scopus_food_subset_metadata.json	data/	JSON	Step 3
wp1_scopus_food_overlap_exact_counts.csv	data/	CSV	Step 4b
wp1_scopus_food_overlap_pairwise.csv	data/	CSV	Step 4b
wp1_scopus_food_overlap_metadata.json	data/	JSON	Step 4b
wp1_scopus_panel_metadata.json	data/	JSON	Step 4a
Fig S1_WP1_Triptych_Scopus_1995_2024.pdf/.png	figures/	PDF+PNG	Step 4a
Fig S2_WP1_FoodOverlap_Scopus_1995_2024.pdf/.png	figures/	PDF+PNG	Step 4b
Table_S1 through Table_S5 (12 files)	supplement/	CSV + XLSX	Step 5
audit_corpus_flow_step01.csv	audit/	CSV	Step 1
audit_build_master.json	audit/	JSON	Step 1
audit_boundary_filter_summary.csv	audit/	CSV	Step 1
audit_input_files.csv	audit/	CSV	Step 1
audit_application_cluster_counts.csv	audit/	CSV	Step 2
audit_application_qc_summary.csv	audit/	CSV	Step 2
audit_display_class_counts.csv	audit/	CSV	Step 2
audit_other_reason_counts.csv	audit/	CSV	Step 2
audit_applied_other_bucket_counts.csv	audit/	CSV	Step 2
wp1_scopus_food_other_bucket_totals.csv	data/	CSV	Step 3
wp1_scopus_food_other_bucket_detail_totals.csv	data/	CSV	Step 3
