# Supplementary material for: From Microalgal Biomass to Products: Downstream Processing Technology Gaps and the Road to Commercial Diversification
Source: Microorganisms. 2026 Jun 24;14(7):1393. doi: 10.3390/microorganisms14071393 (PMC13414295; doi:10.3390/microorganisms14071393)
Supplement: Supplementary file 1 [file microorganisms-14-01393-s001.zip › microorganisms-4336140-supplementary S2.pdf]

Supplementary S2. Food-blocker sensitivity and manual validation

Supplementary S2 evaluates the stability of the food-domain bibliometric results in response to two reviewer concerns: whether the food-blocker rule set affects the food-processing estimate, and whether it suppresses food-wastewater or food-biofuel co-classification. All analyses use the final 1995-2025 Scopus corpus. We first compare four food-blocker settings, then audit all 55 blocker terms by leave-one-out removal and finally report a two-rater manual validation of a stratified 360-record sample. Section S2.1 reports the scenario sensitivity analysis, Section S2.2 reports the leave-one-out audit, Section S2.3 reports food-subtopic stability, and Section S2.4 reports manual validation.

Methods for Supplementary S2

Corpus and classification input

The sensitivity and validation analyses used the same final Scopus bibliometric corpus as Section 2. The corpus was restricted to complete publication years, 1995-2025 and contained 67,195 retained journal articles and reviews after the microalgae/cyanobacteria boundary filter. The applied-domain classifier used title, author keywords, and abstract text. The food-domain sensitivity analysis did not alter the corpus, boundary filter, deduplication or non-food classification rule sets; only the food-domain blocker list was changed.

Food-domain blocker scenarios

The current food-domain classifier used a 55-term blocker list to prevent records with strong non-food domain signals from entering the clean food-primary subset. Three sensitivity settings were generated from this classifier. In the targeted setting, only wastewater/remediation and biofuel/bioenergy blocker terms were removed, leaving 36 terms. In the conservative relaxed setting, only the strongest wastewater, biofuel, and clinical exclusion terms were retained, leaving 22 terms. In the removed setting, no food blocker was applied. The targeted setting was included as the direct test of whether the zero food-wastewater and food-biofuel overlap was caused by the corresponding blocker terms.

For each setting, the full 1995-2025 corpus was reclassified. The food-processing metric was calculated within the clean food-primary subset as the sum of extraction/fractionation/biorefinery records and processing/downstream/drying records divided by clean food-primary records. Food-wastewater and food-biofuel co-classification was calculated in the broader Food-linked population, defined as records assigned to Food in the multi-label applied-domain field, the primary applied-domain field or the clean primary applied-domain field.

Leave-one-out blocker audit

A leave-one-out audit was performed for all 55 current food-blocker terms. In each run, one blocker term was removed while the remaining 54 terms were retained. The full corpus was reclassified for each single-term removal. For each run, we recorded the number of clean food-primary records, the broader Food-linked population, the combined extraction/fractionation/biorefinery plus processing/downstream count and share, Food-wastewater and food-biofuel co-

classifications, newly food-linked records, newly food-primary-clean records, and newly admitted food-wastewater or food-biofuel overlap records.

Manual-validation sample

A stratified validation set of 360 records was drawn from the blocker-sensitivity audit. The strata were: 50 current clean food-primary records, 50 records gained by targeted wastewater / biofuel blocker loosening, 50 records gained by conservative blocker relaxation, 80 records admitted as wastewater / biofuel overlap under complete blocker removal, 80 records admitted as wastewater / biofuel overlap in the leave-one-out audit and 50 records randomly selected from records not linked to Food under either the current or removed blocker settings.

Two raters independently assigned each record to one of five labels: clean_food, ww_biofuel_crossdomain, nonfood_leakage, ambiguous or exclude. The label clean_food was used for human food ingredients, food products, formulation, sensory, nutrition, digestibility, safety or food-processing records. The label ww_biofuel_crossdomain was used when a record had substantive food or food-sector relevance and belonged to wastewater / remediation or biofuel / bioenergy. The label nonfood_leakage was used for wastewater, biofuel, animal-feed, ecological food-web, pharmaceutical, water-quality or materials records in which food-language was incidental or did not indicate human-food relevance. Rows marked include_for_κ = yes and carrying valid labels from both raters were included in inter-rater analysis. Percentage agreement was calculated as the fraction of included records for which both raters assigned the same label. Cohen's κ was calculated across the five-label scheme. Disagreements were retained for adjudication and are reported separately.

S2.1 Food-blocker scenario sensitivity

The combined extraction/fractionation/biorefinery plus processing/downstream share was similar across blocker settings: 9.3%, 9.0%, 9.8%, and 9.6% for the current, targeted, relaxed, and removed settings, respectively. The total range was 0.77 percentage points. Overlap counts behaved differently. The current and conservative relaxed settings gave no Food-wastewater or food-biofuel co-classifications, whereas targeted loosening introduced 377 Food-wastewater and 355 food-biofuel co-classifications. Complete blocker removal introduced 518 and 507, respectively. We therefore treat the zero-overlap result as a clean-rule-set outcome, not as evidence that cross-domain literature is absent.

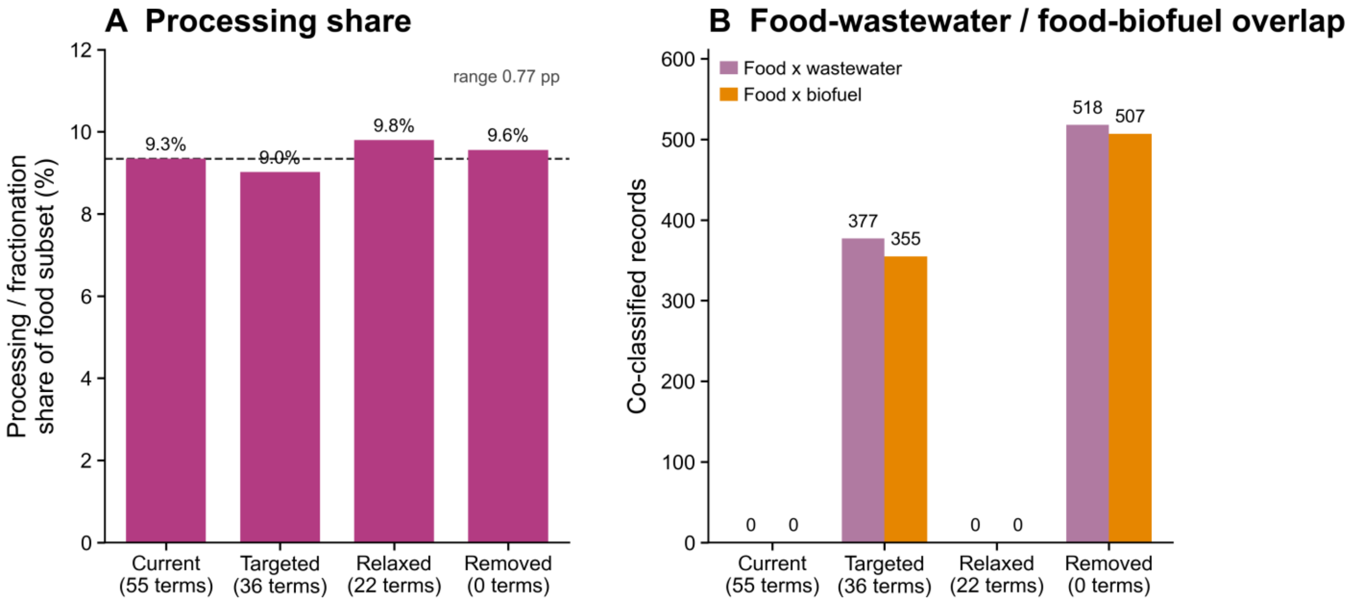

Figure S2.1. **Food-blocker scenario sensitivity.** (A) Combined extraction/fractionation/biorefinery plus processing/downstream share within the clean food-primary subset under four blocker settings. (B) Food-wastewater and food-biofuel co-classification counts under the same settings. The processing/fractionation share remained stable across settings, whereas wastewater/biofuel co-classifications appeared when the corresponding blocker terms were loosened or removed. Underlying data are provided in Supplementary Data S2, Table S2.1.

Table S2.1. Food-blocker scenario sensitivity. Food-domain classification results under four blocker settings in the 1995-2025 Scopus corpus. The table reports the blocker size, clean food-primary records, Food-linked records, extraction/fractionation/biorefinery and processing/downstream counts and shares, and food-wastewater and food-biofuel co-classification counts.

Setting	Blocker terms (n)	Food records (primary-clean)	Food-linked records	Extraction / fract. / bioref. (n)	Extraction / fract. / bioref. (%)	Processing / downstream (n)	Processing / downstream (%)	Combined processing / fract. (n)	Combined processing / fract. (%)	Food × wastewater (n)	Food × biofuel (n)
Current (55 terms)	55	1359	1465	79	5.8	48	3.5	127	9.3	0	0
Targeted (36 terms)	36	1441	2056	81	5.6	49	3.4	130	9.0	377	355
Relaxed (22 terms)	22	1572	1950	95	6.0	59	3.8	154	9.8	0	0
Removed (0 terms)	0	1705	2845	102	6.0	61	3.6	163	9.6	518	507

S2.2 Leave-one-out blocker audit

The leave-one-out audit tested all 55 current food-blocker terms individually. Across all single-term removals, the combined processing/fractionation share remained between 9.20% and 9.58%, with a maximum absolute shift of 0.23 percentage points relative to the current classifier. Thirteen terms generated any new food-wastewater or food-biofuel overlap. The largest effects were observed for wastewater, htl, biofuel, water treatment, and biodiesel.

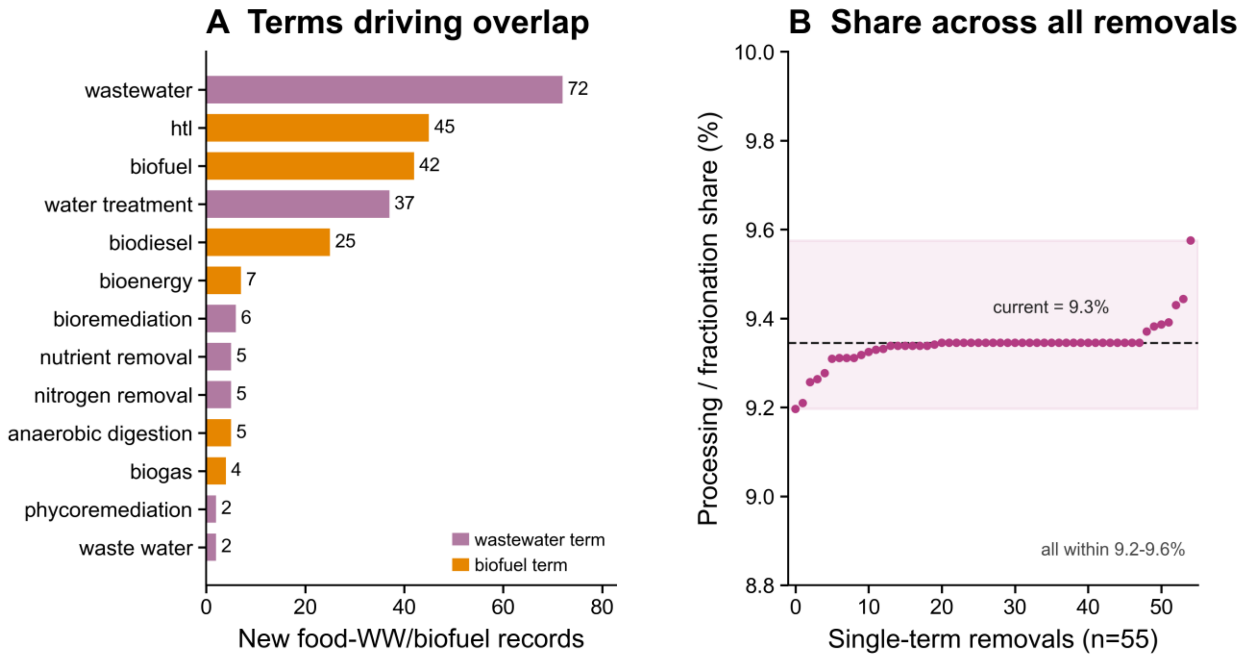

Figure S2.2. Leave-one-out audit of food-blocker terms. (A) Individual blocker terms that introduced food-wastewater or food-biofuel co-classifications when removed one at a time from the current 55-term blocker. (B) Combined extraction/fractionation/biorefinery plus processing/downstream share across all 55 single-term removals. All values remained within 9.20-9.58%, with a maximum absolute shift of 0.23 percentage points relative to the current classifier. Underlying data are provided in Supplementary Data S2, Table S2.2.

Table S2.2. Leave-one-out food-blocker audit. Single-term leave-one-out audit of the current 55-term food-domain blocker. Each row reports the effect of removing one blocker term while retaining the other 54 terms. This table identifies which individual blocker terms drive food-wastewater or food-biofuel overlap while confirming that the food-processing share remains stable across single-term removals.

Left-out term	Food records (primary-clean)	Food-linked records	Combined processing / fract. (n)	Combined processing / fract. (%)	delta share vs current (pp)	Food × wastewater (n)	Food × biofuel (n)	Newly admitted food-WW / biofuel records
wastewater	1372	1537	127	9.26	-0.09	72	0	72
htl	1379	1510	127	9.21	-0.14	0	45	45
biofuel	1381	1507	129	9.34	-0.00	0	42	42
water treatment	1360	1502	127	9.34	-0.01	37	0	37
biodiesel	1364	1490	127	9.31	-0.03	0	25	25
bioenergy	1364	1472	127	9.31	-0.03	0	7	7
bioremediation	1362	1471	127	9.32	-0.02	6	0	6
nitrogen removal	1359	1470	127	9.35	0.00	5	0	5
anaerobic digestion	1359	1470	127	9.35	0.00	0	5	5
nutrient removal	1359	1470	127	9.35	0.00	5	0	5
biogas	1359	1469	127	9.35	0.00	0	4	4
waste water	1359	1467	127	9.35	0.00	2	0	2
phycoremediation	1361	1467	127	9.33	-0.01	2	0	2
bioethanol	1359	1465	127	9.35	0.00	0	0	0
clinical trial	1359	1465	127	9.35	0.00	0	0	0
composite	1368	1486	129	9.43	0.08	0	0	0
aquaculture	1381	1503	127	9.20	-0.15	0	0	0
biolubricant	1359	1465	127	9.35	0.00	0	0	0
bioplastic	1360	1467	127	9.34	-0.01	0	0	0
broiler	1359	1470	127	9.35	0.00	0	0	0
broodstock	1359	1465	127	9.35	0.00	0	0	0
clinical	1371	1500	127	9.26	-0.08	0	0	0
anticancer	1372	1492	128	9.33	-0.02	0	0	0
feed supplement	1359	1470	127	9.35	0.00	0	0	0
feed additive	1359	1475	127	9.35	0.00	0	0	0
engine	1389	1496	133	9.58	0.23	0	0	0
drug delivery	1359	1468	127	9.35	0.00	0	0	0
cutaneous	1360	1469	127	9.34	-0.01	0	0	0
cosmetic	1366	1478	129	9.44	0.10	0	0	0
cosmeceutical	1360	1466	127	9.34	-0.01	0	0	0
larval diet	1359	1465	127	9.35	0.00	0	0	0
lubricant	1359	1466	127	9.35	0.00	0	0	0
livestock	1369	1485	127	9.28	-0.07	0	0	0
live feed	1359	1465	127	9.35	0.00	0	0	0
personal care	1359	1466	127	9.35	0.00	0	0	0

pharmaceutical	1375	1499	128	9.31	-0.04	0	0	0
gasification	1359	1465	127	9.35	0.00	0	0	0
hydrogel	1363	1470	127	9.32	-0.03	0	0	0
hydrothermal liquefaction	1359	1465	127	9.35	0.00	0	0	0
poultry	1364	1481	127	9.31	-0.03	0	0	0
polymer	1385	1505	130	9.39	0.04	0	0	0
phosphorus removal	1359	1465	127	9.35	0.00	0	0	0
protothecosis	1360	1471	127	9.34	-0.01	0	0	0
shrimp	1359	1472	127	9.35	0.00	0	0	0
pyrolysis	1359	1465	127	9.35	0.00	0	0	0
resin	1363	1474	128	9.39	0.05	0	0	0
salmon	1366	1479	128	9.37	0.03	0	0	0
sunscreen	1359	1465	127	9.35	0.00	0	0	0
skincare	1359	1465	127	9.35	0.00	0	0	0
skin care	1359	1465	127	9.35	0.00	0	0	0
therapeutic	1375	1495	129	9.38	0.04	0	0	0
tribological	1360	1466	127	9.34	-0.01	0	0	0
transesterification	1359	1465	127	9.35	0.00	0	0	0
tilapia	1359	1466	127	9.35	0.00	0	0	0
tribology	1359	1465	127	9.35	0.00	0	0	0

Supplementary S2 - Food-blocker sensitivity and manual validation

Figure S2.3 Food-subtopic stability

Food-subtopic composition was compared across the same four blocker settings. The major subtopics remained similar across settings, including the processing-related categories used in the sensitivity analysis.

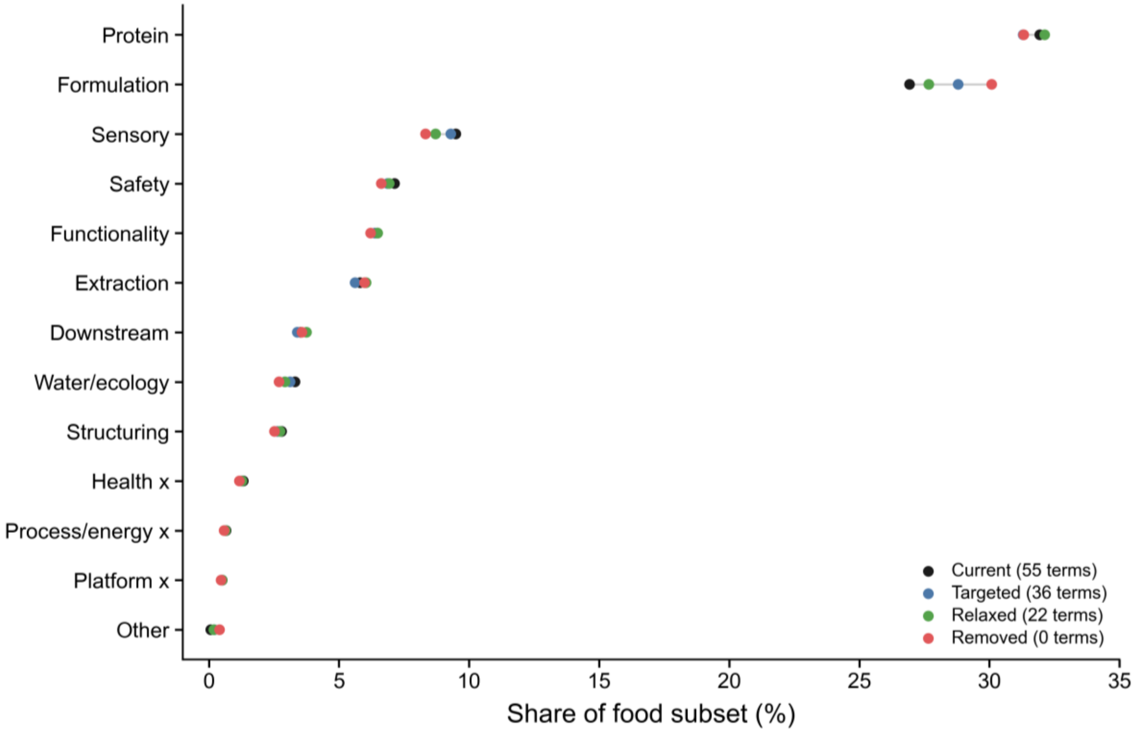

Figure S2.3. Food-subtopic composition across blocker settings. Food-subtopic shares within the clean food-primary subset under the current, targeted, relaxed, and removed blocker settings. Major subtopics remained similar across settings, including the extraction/fractionation/biorefinery and processing/downstream categories. Underlying data are provided in Supplementary Data S2, Table S2.4.

Table S2.3. Food-blocker term sets and scenario definitions. Terms in the current 55-term food-domain blocker and their source-domain assignment. Terms marked "yes" were removed in the targeted wastewater/biofuel-loosening scenario. The same 55 terms were tested individually in the leave-one-out audit.

Blocker term	Source domain	Removed in targeted setting (WW/biofuel)
anaerobic digestion	Biofuels / bioenergy	yes
biodiesel	Biofuels / bioenergy	yes
bioenergy	Biofuels / bioenergy	yes
bioethanol	Biofuels / bioenergy	yes
biofuel	Biofuels / bioenergy	yes
biogas	Biofuels / bioenergy	yes
gasification	Biofuels / bioenergy	yes
htl	Biofuels / bioenergy	yes
hydrothermal liquefaction	Biofuels / bioenergy	yes
pyrolysis	Biofuels / bioenergy	yes
transesterification	Biofuels / bioenergy	yes
biolubricant	Biomaterials / bioplastics / circular economy	-
bioplastic	Biomaterials / bioplastics / circular economy	-
composite	Biomaterials / bioplastics / circular economy	-
hydrogel	Biomaterials / bioplastics / circular economy	-
lubricant	Biomaterials / bioplastics / circular economy	-
polymer	Biomaterials / bioplastics / circular economy	-
resin	Biomaterials / bioplastics / circular economy	-
tribological	Biomaterials / bioplastics / circular economy	-
tribology	Biomaterials / bioplastics / circular economy	-
cosmeceutical	Cosmetics / pigments / colorants	-
cosmetic	Cosmetics / pigments / colorants	-
personal care	Cosmetics / pigments / colorants	-
skin care	Cosmetics / pigments / colorants	-
skincare	Cosmetics / pigments / colorants	-
sunscreen	Cosmetics / pigments / colorants	-
engine	Extra (combustion / engine context)	-
aquaculture	Feed / aquaculture	-
broiler	Feed / aquaculture	-
broodstock	Feed / aquaculture	-
feed additive	Feed / aquaculture	-
feed supplement	Feed / aquaculture	-
larval diet	Feed / aquaculture	-
live feed	Feed / aquaculture	-
livestock	Feed / aquaculture	-
poultry	Feed / aquaculture	-
salmon	Feed / aquaculture	-
shrimp	Feed / aquaculture	-
tilapia	Feed / aquaculture	-
anticancer	Pharmaceuticals / healthcare	-
clinical	Pharmaceuticals / healthcare	-
clinical trial	Pharmaceuticals / healthcare	-
cutaneous	Pharmaceuticals / healthcare	-
drug delivery	Pharmaceuticals / healthcare	-
pharmaceutical	Pharmaceuticals / healthcare	-
protothecosis	Pharmaceuticals / healthcare	-
therapeutic	Pharmaceuticals / healthcare	-
bioremediation	Wastewater / environmental remediation	yes
nitrogen removal	Wastewater / environmental remediation	yes
nutrient removal	Wastewater / environmental remediation	yes
phosphorus removal	Wastewater / environmental remediation	yes
phycoremediation	Wastewater / environmental remediation	yes
waste water	Wastewater / environmental remediation	yes
wastewater	Wastewater / environmental remediation	yes
water treatment	Wastewater / environmental remediation	yes
Total: 55 terms; 19 wastewater/biofuel terms were removed in the targeted setting.		

Table S2.4. Food-subtopic composition across blocker settings. Food-subtopic counts and shares within the clean food-primary subset under the current, targeted, conservative relaxed and removed blocker settings. The combined extraction/fractionation/biorefinery plus processing/downstream row is included to match the processing-share metric used in Fig. S2.1 and Table S2.1.

Food subtopic	Current (55)	Targeted (36)	Relaxed (22)	Removed (0)
**Protein / nutrition**	434 (31.9%)	451 (31.3%)	505 (32.1%)	534 (31.3%)
**Formulation / product development**	366 (26.9%)	415 (28.8%)	435 (27.7%)	513 (30.1%)
**Functionality / techno-functional**	88 (6.5%)	92 (6.4%)	102 (6.5%)	106 (6.2%)
**Sensory / flavor / odor / color**	129 (9.5%)	134 (9.3%)	137 (8.7%)	142 (8.3%)
**Extraction / fractionation / biorefinery**	79 (5.8%)	81 (5.6%)	95 (6.0%)	102 (6.0%)
**Processing / downstream / drying**	48 (3.5%)	49 (3.4%)	59 (3.8%)	61 (3.6%)
**Safety / digestibility / regulation**	97 (7.1%)	99 (6.9%)	109 (6.9%)	113 (6.6%)
**Texturization / structuring**	38 (2.8%)	38 (2.6%)	43 (2.7%)	43 (2.5%)
**Food-other: water / ecology crossover**	45 (3.3%)	45 (3.1%)	46 (2.9%)	46 (2.7%)
**Food-other: health / bioactivity crossover**	18 (1.3%)	18 (1.2%)	20 (1.3%)	20 (1.2%)
**Food-other: process / energy / materials crossover**	9 (0.7%)	9 (0.6%)	10 (0.6%)	10 (0.6%)
**Food-other: platform crossover**	7 (0.5%)	7 (0.5%)	8 (0.5%)	8 (0.5%)
**Other**	1 (0.1%)	3 (0.2%)	3 (0.2%)	7 (0.4%)
**Extraction + Processing combined**	127 (9.3%)	130 (9.0%)	154 (9.8%)	163 (9.6%)

S2.4 Manual validation

Two raters independently reclassified 360 stratified records. Before adjudication, the raters agreed on 351 of 360 records, corresponding to 97.5% agreement and Cohen's κ = 0.937. Among records with direct agreement, the consensus labels were 66 clean_food, 14 ww_biofuel_crossdomain, 267 nonfood_leakage and 4 exclude.

Table S2.5. Manual-validation agreement summary. Two-rater agreement for the stratified 360-record validation set. Values are shown before adjudication of the 9 disagreements.

Metric	Value
Validated records included for κ	360
Rater agreement	351 / 360
Percent agreement	97.5%
Cohen's κ	0.937
Disagreements pending adjudication	9
Agreed clean_food records	66
Agreed ww_biofuel_crossdomain records	14
Agreed nonfood_leakage records	267
Agreed exclude records	4

Table S2.6. Manual-validation design and per-stratum label summary. Label distribution by validation stratum. Counts are based on records for which both raters assigned the same label. Disagreements are reported separately and were retained for adjudication.

Validation stratum	n	agreement n (%)	clean_food	ww_biofuel_crossdomain	nonfood_leakage	exclude	disagreements	nonfood leakage among agreed (%)
Conservative-relaxation gains	50	46 (92.0%)	18	0	27	1	4	58.7
Targeted WW/biofuel gains	50	50 (100.0%)	3	8	36	3	0	72.0
Leave-one-out WW/biofuel leakage	80	78 (97.5%)	8	4	66	0	2	84.6
Random non-Food-linked records	50	50 (100.0%)	2	0	48	0	0	96.0
Removed-blocker WW/biofuel leakage	80	79 (98.8%)	2	2	75	0	1	94.9
Current clean food-primary records	50	48 (96.0%)	33	0	15	0	2	31.2
